# COVID-19 Surveillance in the Biobank at the Colorado Center for Personalized Medicine: Observational Study

**DOI:** 10.2196/37327

**Published:** 2022-06-13

**Authors:** Randi K Johnson, Katie M Marker, David Mayer, Jonathan Shortt, David Kao, Kathleen C Barnes, Jan T Lowery, Christopher R Gignoux

**Affiliations:** 1 Division of Biomedical Informatics and Personalized Medicine Department of Medicine University of Colorado School of Medicine Aurora, CO United States; 2 Department of Epidemiology Colorado School of Public Health Aurora, CO United States; 3 Colorado Center for Personalized Medicine Aurora, CO United States; 4 Human Medical Genetics and Genomics Program University of Colorado Anschutz Medical Campus Aurora, CO United States

**Keywords:** COVID-19, surveillance, pandemic, biobank, EHR, public health, integrated data, population health, health monitoring, electronic health record, eHealth, health record, emergency response, vaccination status, vaccination, testing, symptom, disease impact

## Abstract

**Background:**

Characterizing the experience and impact of the COVID-19 pandemic among various populations remains challenging due to the limitations inherent in common data sources, such as electronic health records (EHRs) or cross-sectional surveys.

**Objective:**

This study aims to describe testing behaviors, symptoms, impact, vaccination status, and case ascertainment during the COVID-19 pandemic using integrated data sources.

**Methods:**

In summer 2020 and 2021, we surveyed participants enrolled in the Biobank at the Colorado Center for Personalized Medicine (CCPM; N=180,599) about their experience with COVID-19. The prevalence of testing, symptoms, and impacts of COVID-19 on employment, family life, and physical and mental health were calculated overall and by demographic categories. Survey respondents who reported receiving a positive COVID-19 test result were considered a “confirmed case” of COVID-19. Using EHRs, we compared COVID-19 case ascertainment and characteristics in EHRs versus the survey. Positive cases were identified in EHRs using the International Statistical Classification of Diseases, 10th revision (ICD-10) diagnosis codes, health care encounter types, and encounter primary diagnoses.

**Results:**

Of the 25,063 (13.9%) survey respondents, 10,661 (42.5%) had been tested for COVID-19, and of those, 1366 (12.8%) tested positive. Nearly half of those tested had symptoms or had been exposed to someone who was infected. Young adults (18-29 years) and Hispanics were more likely to have positive tests compared to older adults and persons of other racial/ethnic groups. Mental health (n=13,688, 54.6%) and family life (n=12,233, 48.8%) were most negatively affected by the pandemic and more so among younger groups and women; negative impacts on employment were more commonly reported among Black respondents. Of the 10,249 individuals who responded to vaccination questions from version 2 of the survey (summer 2021), 9770 (95.3%) had received the vaccine. After integration with EHR data up to the time of the survey completion, 1006 (4%) of the survey respondents had a discordant COVID-19 case status between EHRs and the survey. Using all longitudinal EHR and survey data, we identified 11,472 (6.4%) COVID-19-positive cases among Biobank participants. In comparison to COVID-19 cases identified through the survey, EHR-identified cases were younger and more likely to be Hispanic.

**Conclusions:**

We found that the COVID-19 pandemic has had far-reaching and varying effects among our Biobank participants. Integrated data assets, such as the Biobank at the CCPM, are key resources for population health monitoring in response to public health emergencies, such as the COVID-19 pandemic.

## Introduction

The COVID-19 global pandemic has caused a significant burden on the health and well-being of our families and communities. It has changed the way we work, socialize, and go about our daily lives. To date, over 888,000 Americans have died from COVID-19, and more than 49 million have been infected with the virus, many of whom have been hospitalized or suffered from a range of symptoms lasting from days to years [1]. Further, the burden of this disease, with respect to infection rates, hospitalizations, deaths, and impacts on physical and mental health, is not evenly distributed throughout the population. Understanding the nature and magnitude of this disease has been challenging due to the evolving nature of this virus, changing recommendations from public health around testing and self-quarantine, and our own health behaviors to avoid exposure.

As we strive to understand this novel virus in terms of risk and outcomes, it is important to assess the impact of COVID-19 among various populations, including those who may experience serious versus mild effects from infection, those who experience symptoms but do not undergo testing, and those who never contract the disease. This broad inquiry requires multiple data sources. Electronic health records (EHRs) are useful for capturing information about persons who seek medical care or become hospitalized due to COVID-19, and thus may reflect more severe cases [2-4]. However, due to incomplete and unstructured data collection in EHRs, self-reported population surveys can provide information about persons with more mild disease who may opt not to seek medical care and those never infected [5]. Combining data sources from EHRs and surveys can mitigate limitations and biases inherent in each as well as optimize capture of the COVID-19 experience in a broader population.

We sought to characterize the experience and impact of the COVID-19 virus among a large and diverse group of persons enrolled in the Biobank at the Colorado Center for Personalized Medicine (CCPM), a collaborative initiative supported by UCHealth and the University of Colorado Anschutz Medical Campus. Specifically, we assessed the prevalence of testing and positive test results, the type and frequency of symptoms, health care utilization, severity of disease, and the impacts of the pandemic on mental and physical health, and employment. Uniquely, for this analysis, we were able to combine clinical data from EHRs with self-reported information collected via an online survey that was offered to all Biobank participants.

We present here results from our analysis of self-reported survey data and clinical data recorded in EHRs for Biobank participants. By combining these unique data sources, we were able to capture more COVID-19-positive cases and assess population differences in symptoms, health care utilization, severity (hospitalization), and personal impact. We also highlight the value of biobanks such as ours in facilitating rapid and comprehensive inquiries about emerging public health threats such as COVID-19.

## Methods

### Study Population

Enrollment in the CCPM Biobank is open to all UCHealth patients who are 18 years of age or older and able to provide consent for themselves through My Health Connection, the mobile EHR patient portal for UCHealth. Enrolled participants consent to use of their clinical data from EHRs and to being recontacted about new research opportunities and to complete surveys. To date, the Biobank has enrolled over 200,000 adult participants from among the 2.5 million UCHealth patients across Colorado. Biobank participants are representative of the whole UCHealth population with respect to age, gender, race/ethnicity, and comorbidity status (Multimedia Appendix 1). For this study, all living Biobank participants with a valid email address were invited to complete an online survey about their experience with the COVID-19 pandemic. Participants were identified by a unique ID generated by Health Data Compass (HDC), the system-wide data warehouse for UCHealth. For this analysis, HDC linked survey responses to participants’ clinical data in EHRs using this unique ID, removed personal identifiers, and deposited the data into a datamart that was accessible to the authors.

### Survey Development and Administration

We developed our survey based on an instrument developed by the International Common Disease Alliance (ICDA) [6] early in the pandemic. Our survey included questions about testing for COVID-19, test results, symptoms related to COVID-19 infection, health care utilization following a positive test or symptoms, underlying health conditions, the impact of COVID-19 on health and well-being, potential household exposure to COVID-19, and current smoking behaviors (Multimedia Appendix 2). Given the novelty of the COVID-19 pandemic, no validated questionnaires were available at the time of our survey development and administration.

We created the survey in REDCap [7], a Health Insurance Portability and Accountability Act of 1996 (HIPAA)-compliant database and research management platform, and created unique survey links for each Biobank participant. Personal invitations to complete the survey were sent by email to all participants beginning in June 2020, with a follow-up reminder to nonresponders within 2 weeks. We repeated the process in October 2020 for all participants newly enrolled between June and October 2020. We revised the survey in March 2021 to include additional questions on vaccine uptake, adverse reactions to the vaccine, and long-term symptoms postinfection (Multimedia Appendix 3). The revised survey was sent to all participants who had not responded to the initial survey and newly enrolled participants through May 2021. In total, survey invites were sent to 180,599 individual participants over the course of 15 months.

### COVID-19 Case and Severity Definitions: Survey and EHRs

A summary of available data and definitions from the EHR and survey is provided in Multimedia Appendix 4. Survey respondents who reported receiving a positive COVID-19 test result were considered a “confirmed case” of COVID-19. Self-reported cases also reported whether the respondent tested positive for COVID-19, saw a doctor in person or through telehealth, visited the emergency room (ER), were hospitalized overnight, stayed home/isolated, or did nothing different. We looked at severity in terms of either hospitalization due to COVID-19 or death after COVID-19. Respondents who reported having 1 or more overnight stays in the hospital were considered to be “hospitalized.”

Positive cases were identified in EHRs using *International Statistical Classification of Diseases, 10th revision* (ICD-10) diagnosis codes, health care encounter types, and encounter primary diagnoses. Participants who received an ICD-10 diagnosis code of U07.1 or at least 1 of 11 COVID-19-specific encounter primary diagnoses (Multimedia Appendix 5) were considered an “EHR-confirmed case.” Participants who were hospitalized in a UCHealth hospital overnight during the 3 days before or up to 21 days after their COVID-19 diagnosis date and who had at least 1 of 64 COVID-19-related encounter primary diagnoses (Multimedia Appendix 6) were considered to be “EHR hospitalized.” To compare positive cases identified from EHRs and the survey, we examined the number of hospitalized cases that were discordant between these data sources.

All-cause mortality data stored in the HDC clinical data warehouse include the cause of death as certified by a physician or coroner/medical examiner, related ICD-10 cause of death codes generated by Centers for Disease Control and prevention (CDC), and age at death. These data are obtained through routine linkage of UCHealth patients with the vital statistics/death certificates provided by the Department of Vital Statistics at the Colorado Department of Public Health and the Environment (CDPHE). Accounting for the ~3-month lag time to register certificates, map ICD-10 cause of death codes, and update the clinical databases, the ascertainment of mortality among UCHealth patients for this analysis is nearly 95% complete.

### Other Definitions

Age and race/ethnicity were determined from EHRs. Race and ethnic indicators were extracted as encoded in EHRs and categorized into 4 racial-ethnic groups to preserve >10 individuals in each group in all analyses, including non-Hispanic White, non-Hispanic Black, any Hispanic, and other.

### Statistical Analysis

We generated descriptive statistics to characterize our study population and responses to survey questions using R version 4.0.5 (R Core Team) [8]. We also stratified respondents with respect to COVID-19 infection status based on reported test status and symptomology. We compared COVID-19-positive individuals who were identified via the survey and via EHRs by demographics and severity (overnight hospitalization and death). We investigated case status and hospitalization misclassification in both the survey and EHRs by comparing those who were discordant in the survey and EHRs. We calculated differences between groups using chi-square and *t* test statistics for categorical and continuous measures, respectively. As expected, due to the large sample size in the study, most comparisons were statistically significant at a 2-sided *α* of <.05. Therefore, we focused results and interpretation on effect sizes and the corresponding SE of the estimate.

### Ethical Considerations

The Colorado Multiple Institutional Review Board (COMIRB) approved all CCPM Biobank study protocols (COMIRB #15-0461), and this research was performed in accordance with relevant guidelines/regulations.

## Results

### Survey Response

**Figure 1:**
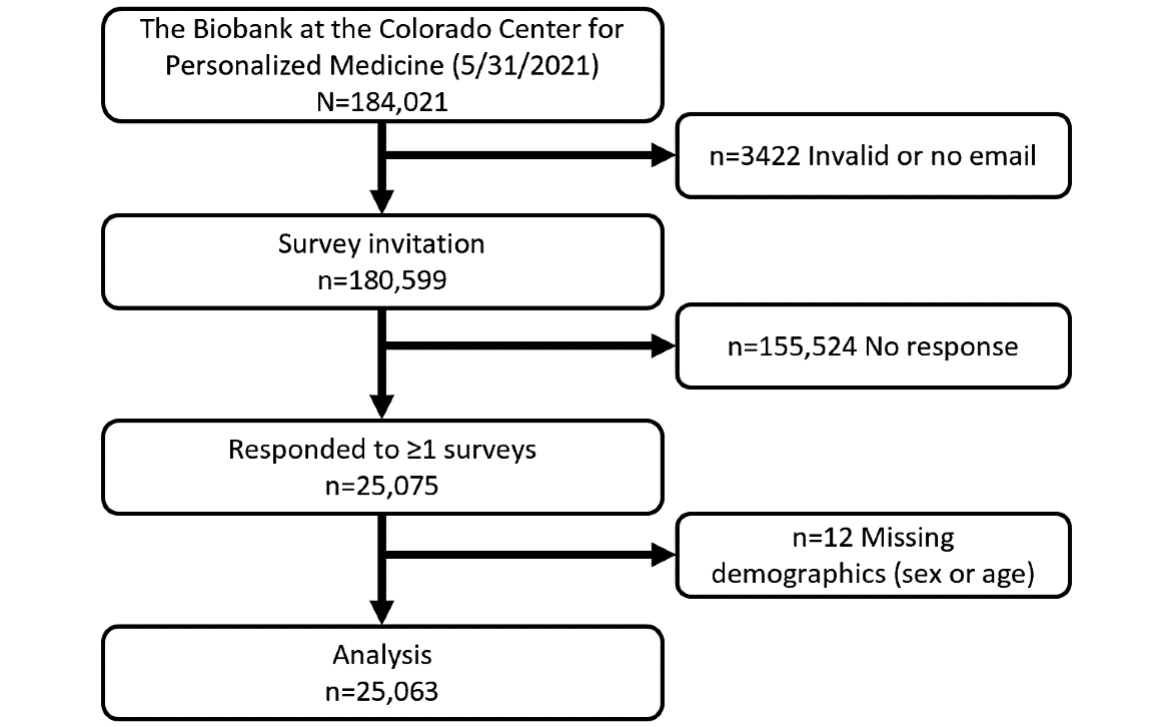
The CCPM Biobank COVID-19 survey population. CCPM: Colorado Center for Personalized Medicine.

Of 180,599 Biobank participants with valid email addresses, 25,063 (13.9% response rate) completed at least 1 survey and had complete demographic information (Figure 1). Compared to nonrespondents (Multimedia Appendix 7), respondents were older (mean age 55.0 years vs 48.6 years, *P*<.001) and enriched for a higher proportion of females (n=15,695, 62.6%, vs n=91,707, 59.0%, *P*<.001) and individuals of non-Hispanic White race/ethnicity (n=21,917, 87.4%, vs n=119,848, 77.1%, *P*<.001).

### COVID-19 Testing

Among all survey respondents, 10,661 (42.5%) reported being tested for COVID-19. The most common reasons for testing were having symptoms (n=3148, 29.5%), exposure to someone who tested positive for COVID-19 (n=1975, 18.5%), doctor recommendation (n=1565, 14.7%), requirement of the employer (n=950, 8.9%), and recent international travel (n=362, 3.4%). An additional 4352 (40.8%) of individuals tested reported other reasons for testing that included having surgery or other medical procedure, planned travel, a desire or need to be around large groups or family members, and work site offerings for testing.

Of those tested, 1366 (12.8%) tested positive for COVID-19 (Table 1) and were considered confirmed cases. The distributions of age, sex, race/ethnicity, college education, number of symptoms, number of preexisting comorbidities, overall health status, and exposure to a household member who tested positive for COVID-19 were different across the 3 groups of those who tested positive, tested negative, and were not tested (all *P*<.001). Young adults (aged 18-29 years) were overrepresented among the tested-positive group, representing 146 (10.7%) of those who tested positive compared to 619 (6.7%) of those who tested negative and 738 (5.1%) of those who were not tested (*P* for trend <.001). Similarly, individuals of Hispanic race/ethnicity were overrepresented in the tested-positive group at 125 (9.2%) compared to 528 (5.7%) of those who tested negative and 619 (4.3%) of those who were not tested. Individuals who tested positive were also more likely to report symptoms, household exposure to COVID-19, and poor health status (Table 1; all *P*<.001).

**Table 1:** COVID-19 testing in the Biobank among survey respondents.

Characteristics			Total respondents (N=25,063)		Tested (N=10,661)								Not tested (N=14,402)								*P* value^a^			
					Tested positive (N=1366)		Tested negative (N=9295)		*P* value^b^			Respondents				*P* value^c^								
Age (years), mean (SD)			55.0 (15.8)		48.9 (14.6)		53.7 (15.6)		<.001			56.5 (15.8)				<.001			<.001					
**Age (years), n (%)**										<.001				N/A^d^			<.001			<.001				
	18-29	1503 (6.0)		146 (10.7)		619 (6.7)		N/A			738 (5.1)				N/A			N/A						
	30-64	15,049 (60.0)		1000 (73.2)		5890 (63.4)		N/A			8159 (56.7)				N/A			N/A						
	65+	8511 (34.0)		220 (16.1)		2786 (30.0)		N/A			5505 (38.2)				N/A			N/A						
**Sex, n (%)**										.09				N/A			<.001			<.001				
	Female	15,695 (62.6)		902 (66.0)		5915 (63.6)		N/A			8878 (61.6)				N/A			N/A						
	Male	9368 (37.4)		464 (34.0)		3380 (36.4)		N/A			5524 (38.4)				N/A			N/A						
**Race/ethnicity, n (%)**										<.001				N/A			<.001			<.001				
	Non-Hispanic White	21,916 (87.4)		1117 (81.8)		8072 (86.8)		N/A			12,727 (88.4)				N/A			N/A						
	Non-Hispanic Black	308 (1.2)		24.0 (1.8)		133 (1.4)		N/A			151 (1.0)				N/A			N/A						
	Hispanic	1272 (5.1)		125 (9.2)		528 (5.7)		N/A			619 (4.3)				N/A			N/A						
	Other	1567 (6.3)		100 (7.3)		562 (6.0)		N/A			905 (6.3)				N/A			N/A						
**Bachelor’s degree, n (%)**										<.001							.13			<.001				
	Yes	19407 (77.4)		973 (71.2)		7219 (77.7)		N/A			11,215 (77.9)				N/A			N/A						
	No	5482 (21.9)		381 (27.9)		2008 (21.6)		N/A			3093 (21.5)				N/A			N/A						
	Unknown	174 (0.7)		12.0 (0.9)		68.0 (0.7)		N/A			94.0 (0.7)				N/A			N/A						
Number of acute symptoms, mean (SD)			0.261 (1.05)		2.09 (2.61)		0.393 (1.15)		<.001			0.00222 (0.0897)				<.001			<.001					
Number of comorbidities, mean (SD)			1.51 (1.38)		1.46 (1.46)		1.59 (1.44)		.004			1.46 (1.32)				<.001			<.001					
**Health status, n (%)**										<.001				N/A			<.001			<.001				
	Excellent	5664 (22.6)		235 (17.2)		1993 (21.4)		N/A			3436 (23.9)				N/A			N/A						
	Very good	10,532 (42.0)		444 (32.5)		3784 (40.7)		N/A			6304 (43.8)				N/A			N/A						
	Good	6558 (26.2)		440 (32.2)		2527 (27.2)		N/A			3591 (24.9)				N/A			N/A						
	Fair	1859 (7.4)		196 (14.3)		793 (8.5)		N/A			870 (6.0)				N/A			N/A						
	Poor	323 (1.3)		45.0 (3.3)		151 (1.6)		N/A			127 (0.9)				N/A			N/A						
	Unknown	127 (0.5)		6.00 (0.4)		47.0 (0.5)		N/A			74.0 (0.5)				N/A			N/A						
**Questionnaire version, n (%)**										<.001				N/A			<.001			<.001				
	1-Summer-fall 2020	14,814 (59.1)		410 (30.0)		3718 (40.0)		N/A			10,686 (74.2)				N/A			N/A						
	2-Summer-fall 2021	10,249 (40.9)		956 (70.0)		5577 (60.0)		N/A			3716 (25.8)				N/A			N/A						
**EHR^e^ COVID-19 case, n (%)**										<.001				N/A			<.001			<.001				
	Yes	717 (2.9)		519 (38.0)		125 (1.3)		N/A			73.0 (0.5)				N/A			N/A						
	No	24,346 (97.1)		847 (62.0)		9170 (98.7)		N/A			14,329 (99.5)				N/A			N/A						
**Household member tested positive, n (%)**										<.001				N/A			<.001			<.001				
	No	19,472 (77.7)		482 (35.3)		7268 (78.2)		N/A			11,722 (81.4)				N/A			N/A						
	Yes	1519 (6.1)		691 (50.6)		517 (5.6)		N/A			311 (2.2)				N/A			N/A						
	Unknown	4072 (16.2)		193 (14.1)		1510 (16.2)		N/A			2369 (16.4)				N/A			N/A						
**Genetic data, n (%)**										.17				N/A			<.001			<.001				
	No	20,182 (80.5)		1157 (84.7)		7732 (83.2)		N/A			11,293 (78.4)				N/A			N/A						
	Yes	4881 (19.5)		209 (15.3)		1563 (16.8)		N/A			3109 (21.6)				N/A			N/A						

^a^From chi-square or ANOVA, comparing tested positive versus tested negative versus not tested.

^b^From chi-square or ANOVA, comparing tested positive versus tested negative.

^c^From chi-square or ANOVA, comparing tested versus not tested.

^d^N/A: not applicable.

^e^EHR: electronic health record.

### COVID-19 Case Symptomology

**Figure 2:**
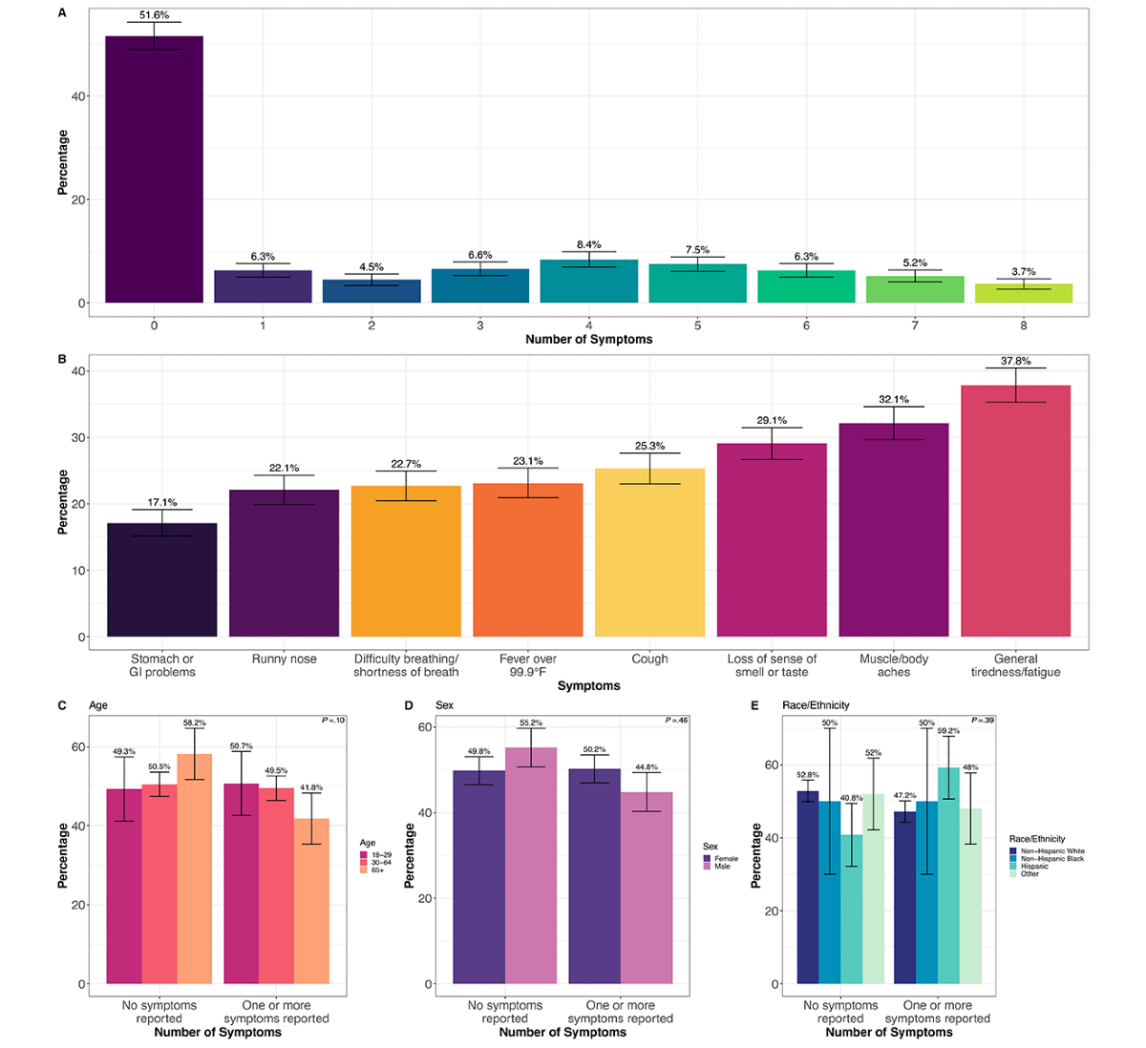
Symptomology among COVID-19 cases. Each symptom was reported 14 days before or 14 days after a positive COVID-19 test. (A) Number of symptoms reported among COVID-19 cases. (B) Percentage of COVID-19-positive cases that reported each symptom. Comparing asymptomatic cases with symptomatic cases (at least 1 symptom) by (C) age, (D) sex, and (E) race/ethnicity. *P* value from the Pearson chi-square test for different distributions across demographic groups. Error bars indicate the 95% CI for the percentage point estimate. GI: gastrointestinal.

Of the 1366 COVID-19-positive individuals identified from the survey, 1154 (84.4%) individuals had at least 1 of the following COVID-19-related symptoms since February 2020: cough, fever over 99.9°F, general tiredness/fatigue, muscle/body aches, runny nose, difficulty breathing/shortness of breath, loss of sense of smell or taste, and stomach or gastrointestinal (GI) problems (Figure 2). However, only 661 (48.4%) reported at least 1 symptom 14 days before or after a positive COVID-19 test. The number of symptoms individuals reported was relatively even from 1 to 8 symptoms, ranging from 50 (3.7%) individuals reporting all 8 symptoms and 115 (8.4%) reporting 4 symptoms (Figure 2A). General tiredness/fatigue and muscle/body aches were the most commonly reported symptoms within 14 days of a positive COVID-19 test, at 517 (37.8%) and 439 (32.1%) individuals, respectively (Figure 2B). The next most common symptom was loss of sense of smell or taste, with 397 (29.1%) individuals reporting within 14 days of a positive COVID-19 test (Figure 2B). However, an additional 283 (20.7%) individuals reported this symptom outside the 28-day window. A quarter of the individuals (n=346, 25.3%) reported a cough within 14 days of a positive COVID-19 test, and 310 (22.7%) and 302 (22.1%) reported difficulty breathing/shortness of breath and a runny nose, respectively (Figure 2B). Only 234 (17.1%) of individuals reported stomach or GI problems (Figure 2B). The remainder (n=705, 51.6%) reported no symptoms within 14 days before or after their COVID-19 positive test. There were no significant differences in asymptomatic cases compared to symptomatic cases (having at least 1 symptom) when comparing by age, sex, or race/ethnicity (Figure 2C-E).

### Health Behaviors and Impact on the Health Care System

**Figure 3:**
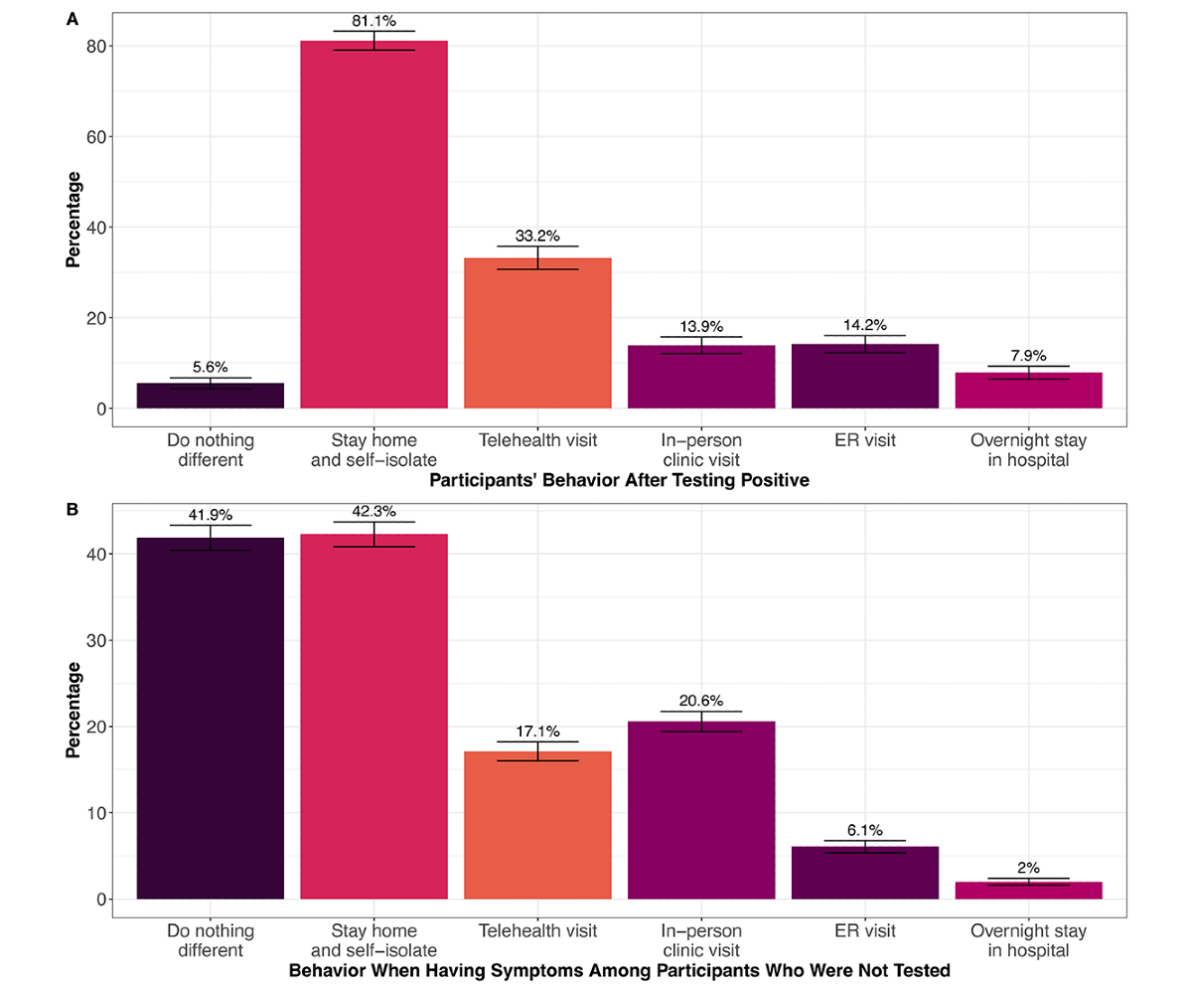
The impact of COVID-19 on the health care system. (A) Participants’ behavior after testing positive for COVID-19 and (B) participants’ behavior when having symptoms, among those with at least 1 symptom who did not get tested for COVID-19. Error bars indicate the 95% CI for the percentage point estimate. ER: emergency room.

To assess health behaviors among COVID-19 cases and the potential impact on the health care system, we asked these individuals what they did as a result of testing positive (Figure 3A). Of the 1366 respondents with positive tests, 1108 (81.1%) stayed home and self-isolated, and 76 (5.6%) did not report any changes in behavior (Figure 3A). Of those who did not change behavior, 63 (82.9%) did not have any symptoms reported 14 days before or after their COVID-19 test. Of the 1366 individuals who tested positive, 625 (45.8%) sought out at least 1 form of medical care: 190 (13.9%) saw a doctor at an in-person visit, 454 (33.2%) saw a doctor via telehealth, 194 (14.2%) went to the ER, and 108 (7.9%) had an overnight stay in a hospital (Figure 3A). A subset of 229 (16.7%) individuals reported being tested at a UCHealth facility versus 213 (15.6%) outside UCHealth, with no response from 924 (67.6%) respondents. Of the 229 (16.7%) respondents who said they tested positive at a UCHealth facility, only 137 (59.8%) were identified as a “case” within EHRs. There was a high rate of missingness for the question on who performed the test (n=924, 67.6%), so there may be confusion by participants about who supplied the COVID-19 test.

Among respondents who were not tested but reported having at least 1 COVID-related symptom, 1901 (41.9%) said they did nothing different, whereas 1920 (42.3%) stayed home and self-isolated (Figure 3B). A third (n=1515, 30.4%) sought out at least 1 form of medical care, 934 (20.6%) had an in-person clinic visit, 77 (17.1%) had a telehealth clinic visit, 275 (6.1%) went to the ER, and 90 (2.0%) had an overnight stay in the hospital (Figure 3B).

### Impact of the COVID-19 Pandemic

The impact of the COVID-19 pandemic on employment, family life, mental health, or physical health was largely negative, with 18,861 (75.3%) of respondents reporting a negative impact from the COVID-19 pandemic in at least 1 of these domains compared to 5856 (23.4%) of respondents reporting a positive impact in at least 1 domain (*P*<.001). Mental health and family life were most negatively affected by the pandemic, at 13,688 (54.6%) and 12,233 (48.8%) of respondents reporting a negative impact, respectively. The negative impact in the other 2 domains was lower at 7059 (28.2%) for physical health and 5320 (21.2%) for employment (*P*<.001).

**Figure 4:**
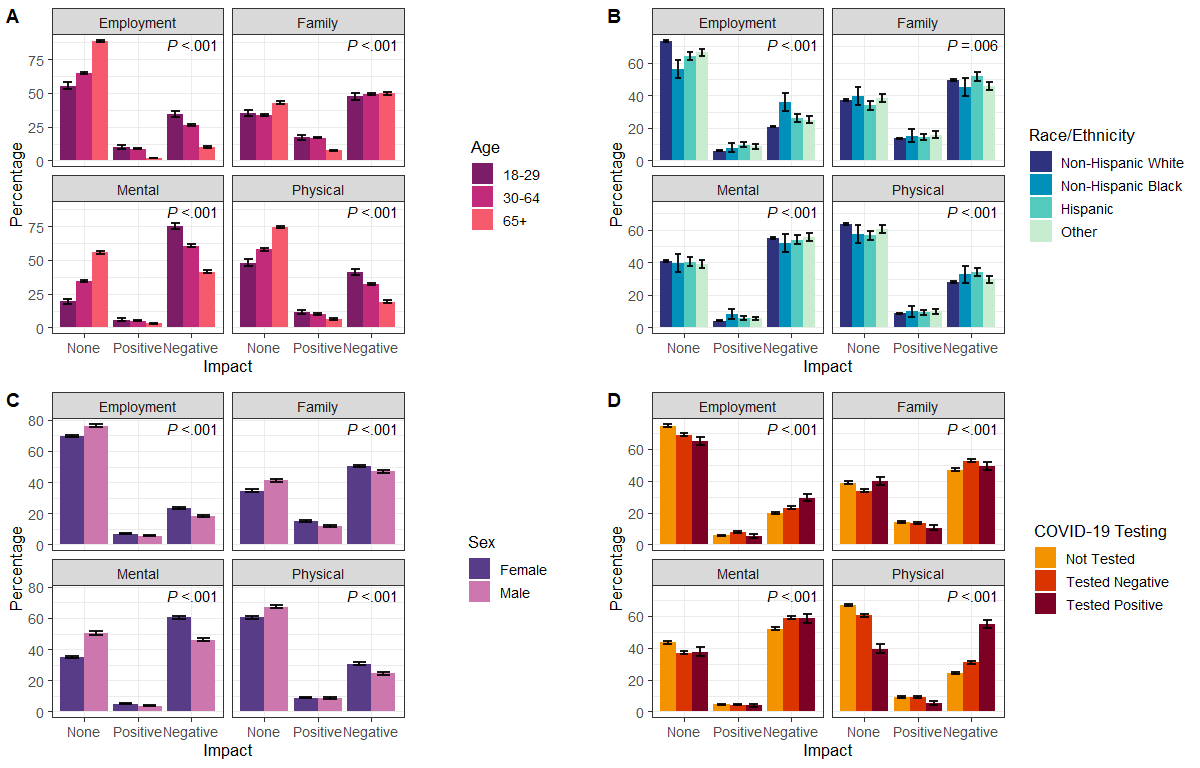
The impact of COVID-19 on employment, family life, and mental and physical health by (A) age, (B) race/ethnicity, (C) sex, and (D) COVID-19 test status. *P* value from the Pearson chi-square test for different distributions across impact and demographic groups. Error bars indicate the 95% CI for the percentage point estimate.

The impact of the COVID-19 pandemic was not equal across groups by age, race/ethnicity, sex, and COVID-19 testing status (maximum *P*=.006; Figure 4). A higher proportion of young adults reported a negative mental health impact (1123/1499, 74.9%, 95% CI 72.7%-77.1%) than adults aged 30-64 years (9092/14,975, 60.7%, 95% CI 59.6%-61.5%) and older adults (65+ years; 3473/8445, 41.1%, 95% CI 40.1%-42.2%). A similar linear trend across age groups was seen for the negative impact of the pandemic on employment and physical health (Figure 4A). Using self-reported race/ethnicity as captured in EHRs, a higher proportion of non-Hispanic Black respondents reported a negative impact on their employment (108/302, 35.8%, 95% CI 30.4%-41.2%) compared to other race/ethnic groups (non-Hispanic White: 4498/21,628, 20.8%; Hispanic: 328/1257, 26.1%; other: 386/1550, 24.9%; Figure 4B). Women reported a greater negative impact of COVID-19 compared to men across all domains: employment (3625/15,470 [23.4%] versus 1695/9267 [18.3%]), family life (7865/15,579 [50.5%] versus 4368/9298 [47.0%]), mental health (9407/15,609 [60.3%] versus 4281/9310 [46.0%]), and physical health (4796/15,600 [30.7%] versus 2263/9293 [24.4%]) (Figure 4C, all *P*<.001). Respondents who tested positive for COVID-19 reported a higher negative impact on their physical health (744/1353, 55.0%, 95% CI 52.3%-57.6%) than those who tested negative (2854/9234, 30.9%, 95% CI 30.0%-31.9%) and those who did not report a COVID-19 test (3461/14,306, 24.2%, 95% CI 23.5%-24.9%, Figure 4D).

### COVID-19 Vaccination

**Figure 5:**
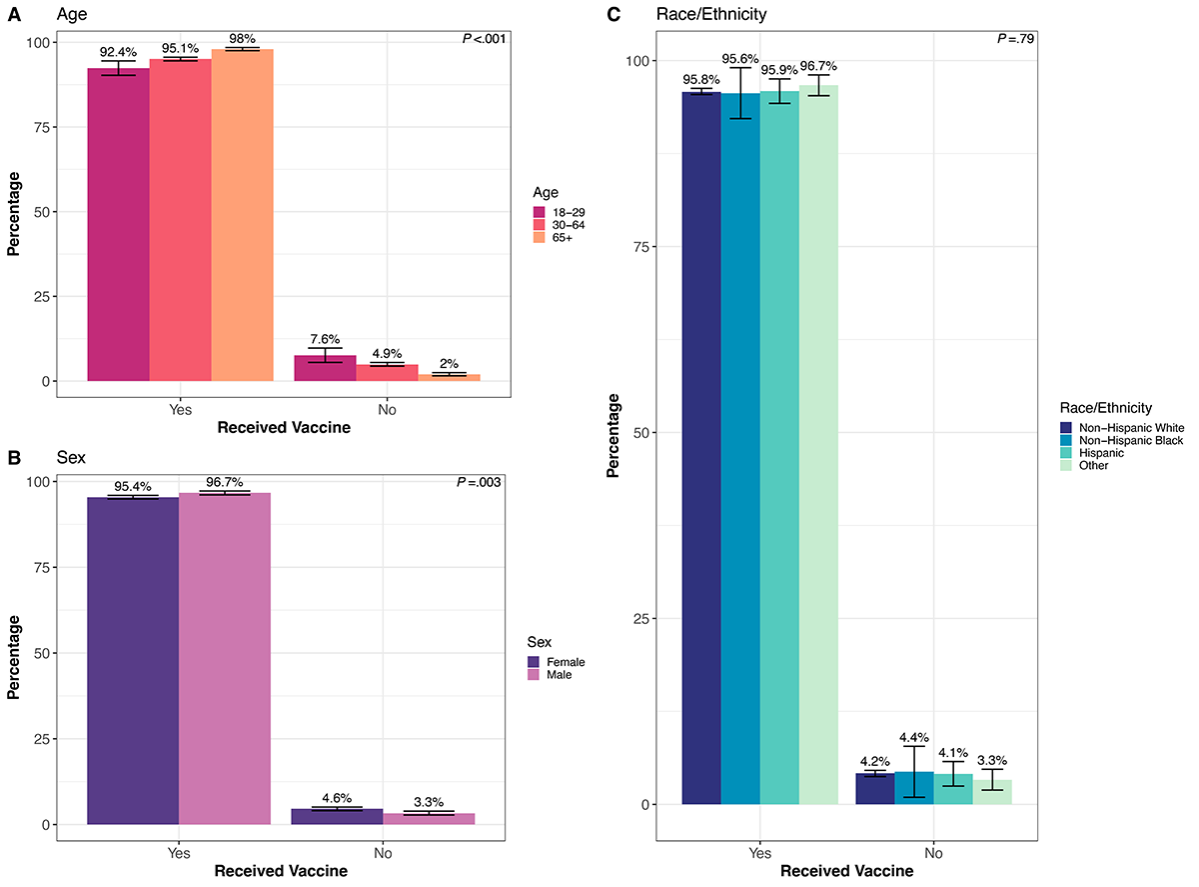
Vaccine uptake by (A) age, (B) sex, and (C) race/ethnicity. *P* value from the Pearson chi-square test for different distributions across impact and demographic groups. Error bars indicate the 95% CI for the percentage point estimate.

In our second round of the survey (administered in spring/summer 2021), we added questions about COVID-19 vaccination. Of the 10,249 (40.9%) of the total overall survey population (N=25,063) who responded to the second survey, 9770 (95.3%) received the vaccine. Younger people were less likely to have received a vaccine: 46 (7.6%) of those aged 18-29 years did *not* receive a vaccine compared to 303 (4.9%) of those aged 30-64 years and 69 (2.0%) of those aged 65+ years (*P*<.001, Figure 5A). Women were slightly less likely to receive a vaccine (n=289, 4.6%, of women vs n=129, 3.3%, of men, *P*=.003, Figure 5B). The vaccination rate was similar across race/ethnicity categories, with 368 (4.2%) non-Hispanic Whites, <10 (4.4%) non-Hispanic Blacks, 23 (4.1%) Hispanics, and 21 (3.3%) in the other race category not receiving vaccines (*P*=.79, Figure 5C). The median income of the home 3-digit zip code was lower for unvaccinated participants: US $67,800 in the unvaccinated compared to US $71,500 in the vaccinated group (*P*<.001). The median percentage of the population that received a bachelor’s degree by 3-digit zip code was lower for unvaccinated (37.7%) compared to vaccinated (47.3%) participants (*P*<.001).

### Demographics of COVID-19 Cases Captured by EHRs vs the Survey

**Figure 6:**
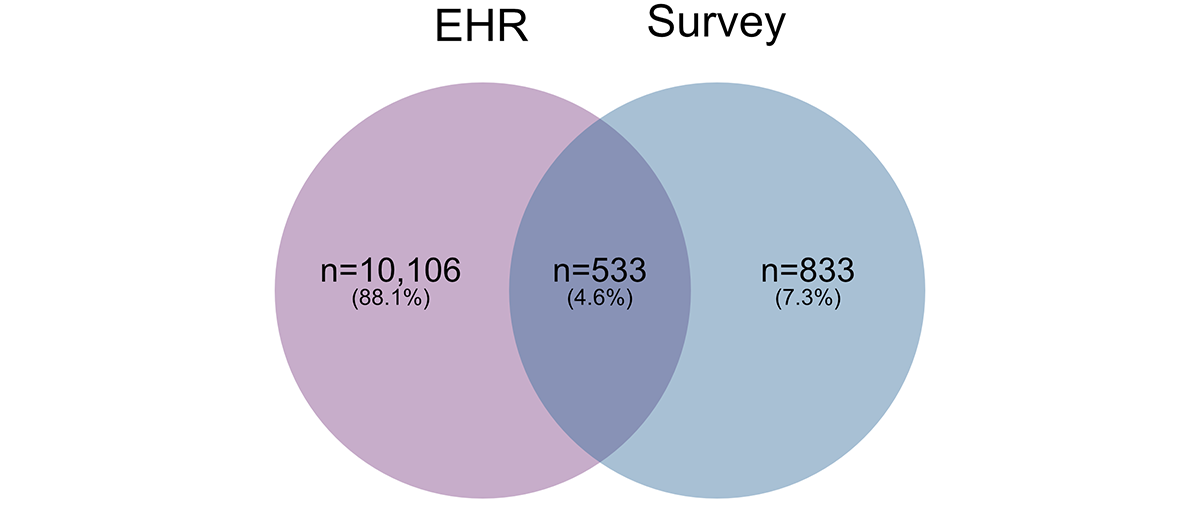
COVID-19-positive CCPM Biobank participants identified through the UCHealth EHRs and the survey. CCPM: Colorado Center for Personalized Medicine; EHR: electronic health record.

We identified 11,472 (6.4%) COVID-19 positive cases from among 180,599 eligible Biobank participants: 1366 (11.9%) from the survey and 10,639 (92.7%) from EHRs; 533 (4.6%) cases were identified in both sources (Figure 6).

**Figure 7:**
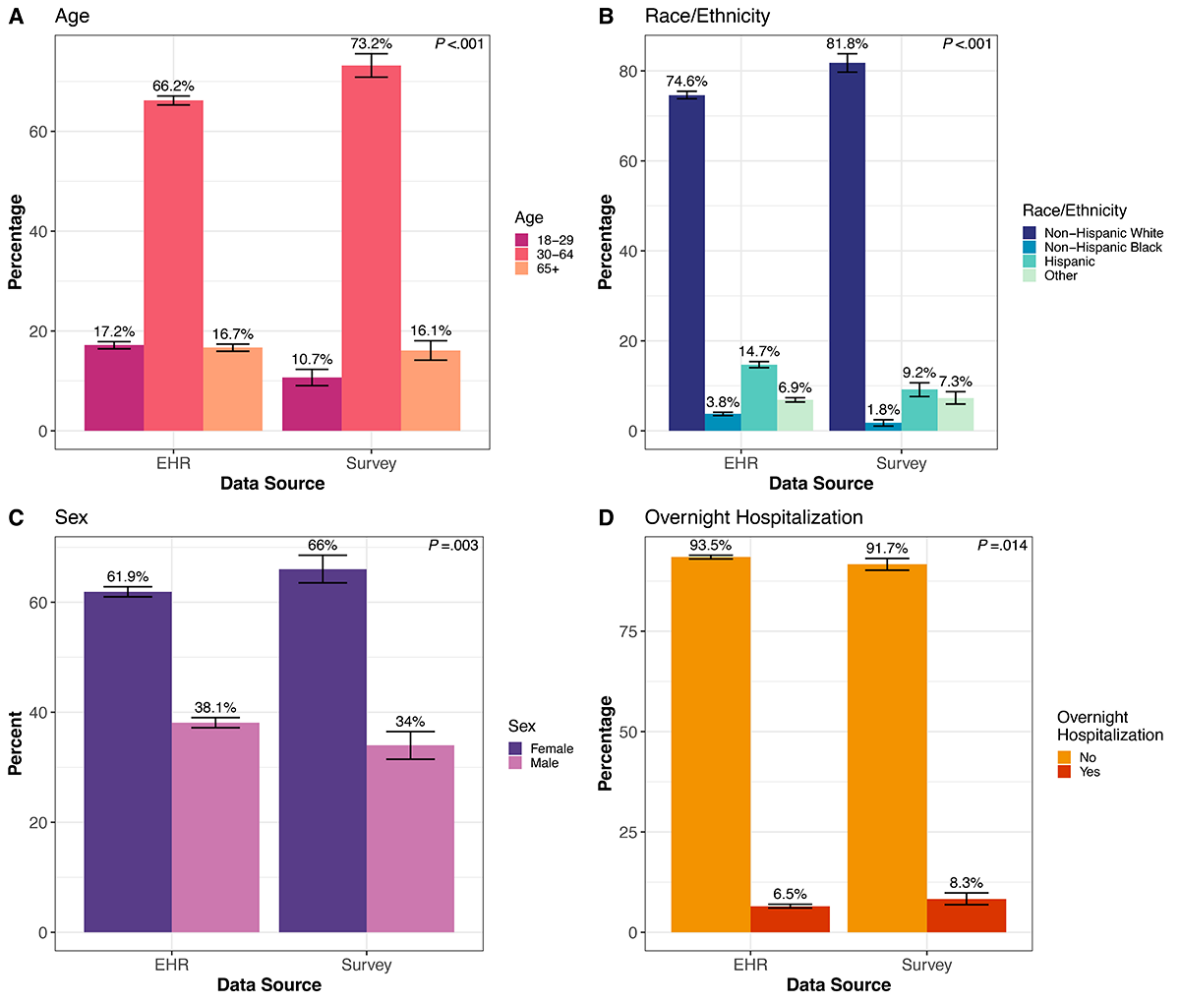
Comparison of COVID-19 cases captured in the EHRs and the survey by (A) age, (B) race/ethnicity, (C) sex, and (D) COVID-19-related overnight hospitalization. *P* value from the Pearson chi-square test for different distributions across impact and demographic groups. Error bars indicate the 95% CI for the percentage point estimate. EHR: electronic health record.

In comparing COVID-19 cases from EHRs to those in the survey (Figure 7), we found that cases identified in EHRs were younger, with 17.2% of individuals in the 18-29 age group compared to 10.7% in the survey group (*P*<.001, Figure 7A). A higher percentage of cases identified in EHRs were Hispanic compared to survey cases (14.7% vs 9.2%, respectively, *P*<.001, Figure 7B). EHR cases also had a slightly lower proportion of women (61.9%) compared to the survey group (66.0%; *P*=.003, Figure 7C). The median income for the 3-digit zip code was the same, US $69,900 in both groups. The median percentage of the population that received a bachelor’s degree by 3-digit zip code was slightly lower in the EHR (41.3%) group compared to the survey (45.7%) group (*P*<.001).

### COVID-19 Case Severity in EHRs and the Survey

A higher percentage of COVID-19-positive cases identified from the survey were hospitalized overnight (8.3%) compared to the EHR (6.5%) group (*P*=.01, Figure 7D). Using all-cause mortality data obtained from CDPHE vital statistics, 130 (2.3%) individuals in the EHR group died, leading to a death rate of 1.2%. In addition, 4 (0.29%) people in the survey group died, with a death rate of 0.2%.

The EHR is a longitudinal data source; therefore, we can capture COVID-19 cases on a continuing basis, whereas the survey reflects a point in time and can only identify individuals who had COVID-19 before they took the survey. Of 907 COVID-19 cases identified in EHRs who completed the survey but did not report a positive COVID-19 diagnosis in the survey, 379 (41.8%) reported receiving a negative COVID-19 test result and 528 (58.2%) had not taken a COVID-19 test and were presumed to be negative. The majority of these individuals (n=732, 80.7%) completed the survey before they were diagnosed with COVID-19 in EHRs.

### COVID-19 Case and Hospitalization Discordance Between EHRs and the Survey

To quantify discordance of the COVID-19 case status between EHRs and the survey, we looked across our entire set of survey respondents (N=25,063). We only counted a participant as “EHR COVID-19 positive” if the diagnosis made was prior to taking the survey, not COVID-19 cases that happened after the survey was taken. Although neither the survey nor EHRs are a gold standard for case classification, we can look at the discordance between them to identify the potential for misclassification. Overall, there were a total of 1006 (4%) respondents discordant for COVID-19 case status. Of the 25,063 individuals who took the survey, 173 (0.7%) were identified as COVID-19 positive in EHRs but negative or not tested in the survey, leading to a discordance rate of 0.7% (Table 2). In addition, 833 (3.3%) individuals were identified as COVID-19 positive in the survey but negative in EHRs, leading to a discordance rate of 3.3%.

To quantify discordance of the hospitalization status in both EHRs and in the survey, we restricted it to individuals who responded to the survey and were COVID-19 positive in either EHRs or the survey (n=2273). EHR hospitalizations were only considered if they were prior to taking the survey. There were 6 (0.3%) individuals who were positive for hospitalization in EHRs but negative in the survey, a discordance rate of 0.3% (Table 3). There were 59 (2.6%) individuals who were positive for hospitalization in the survey who were negative in EHRs, a discordance rate of 2.6%.

**Table 2:** Case status misclassification between the survey and EHRs^a^ (N=25,063).

COVID-19 status	Survey COVID-19 positive, n (%)	Survey COVID-19 other (negative or not tested), n (%)	Total, n (%)
EHR COVID-19 positive	533 (2.1)	173 (0.7)	706 (2.8)
EHR COVID-19 other (negative or not tested)	833 (3.3)	23,524 (93.9)	24,357 (97.2)
Total	1366 (5.5)	23,697 (94.4)	25,063 (100)

^a^EHR: electronic health record.

**Table 3:** Hospitalization misclassification between the survey and EHRs^a^ (N=2273).

Hospitalization	Survey hospitalization positive, n (%)	Survey hospitalization negative, n (%)	Total, n (%)
EHR hospitalization positive	49 (2.2)	6 (0.3)	55 (2.4)
EHR hospitalization negative	59 (2.6)	2159 (95)	2218 (97.6)
Total	108 (4.8)	2165 (95.2)	2273 (100)

^a^EHR: electronic health record.

## Discussion

### Principal Findings

We found that the COVID-19 pandemic has had far-reaching and varying effects among our Biobank participants. Of the 25,063 survey respondents, 10,661 (42.5%) were tested for the virus, 1366 (12.8%) of those tested were positive, and among positive cases, 1154 (84.5%) reported having 1 or more COVID-related symptoms since February 2020 and 625 (45.8%) sought medical care following their diagnosis. The vast majority of all survey respondents (n=18,861, 75%) reported a negative impact from the COVID-19 pandemic—most commonly around mental health and family life. Differences between data captured in EHRs versus those captured in the survey reveal the benefit of using both sources in combination. For example, mild cases with subclinical manifestations of infection that did not result in seeking care may be missing from EHRs but captured in a survey.

### Strengths and Limitations

EHRs are a longitudinal data source that collect clinical information on all patients diagnosed with or treated for COVID-19 within the UCHealth system irrespective of proclivity to participate in research or respond to surveys. As such, EHRs captured COVID-19 cases from Biobank participants that the survey did not. However, a key strength of this study was our ability to leverage an existing, living resource in the CCPM Biobank and survey engine to assess the health and well-being of our participants in ways that are not highlighted by EHRs. Because Biobank participants consent to recontact, we have an opportunity to follow up with subpopulations within our cohort to collect additional information and monitor outcomes such as reinfection and vaccine uptake. Although our overall response to the survey was sizeable, we acknowledge that the composition of the underlying patient population at UCHealth who enrolled in the Biobank and differential responses to the survey may have introduced some bias—results may not generalize outside of the CCPM Biobank and UCHealth population. However, our ability to incorporate EHR data allowed us to build a research population of Biobank participants that is more representative of the entire patient population.

There are benefits and limitations to COVID-19 case ascertainment using either a survey or EHRs. Because both methods of ascertainment draw from the CCPM Biobank, they are both limited to individuals who have sought treatment at a UCHealth facility and enrolled in the CCPM Biobank. Furthermore, the survey is a convenience sample of individuals who responded to an email asking them to participate. The EHR will capture any health care encounter at a UCHealth facility, but it is an open system, and it will not capture all health care encounters for all every Biobank participant. The ascertainment bias in both methods can be a challenge for future analytical studies. We hope that by describing the demographics and case severity in both these methods of collection, future analytical studies will better be able to adjust for these biases.

### Comparison With Prior Work

Our overall case positivity rate of 13% is comparable to those reported by other EHR-based retrospective studies conducted in 2020 and 2021 [9,10]. However, our finding of higher positivity rates (20%) among our younger participants (aged 18-39 years) and Hispanics (19%) has not been reported previously and may reflect differences in reasons for testing in these groups (eg, due to having symptoms or recent exposure vs other reasons). Though not surprising that a large proportion of respondents reported having symptoms, given the breadth of symptoms reported (eg, runny nose, fever, body aches), it is notable that 3026 (34.4%) of those with symptoms did not undergo testing nor seek medical care. It is likely that a percentage of this group had COVID-19 and would not be counted as such via public health surveillance efforts, which could lead to substantial underestimates of the true infection rate in the general population.

We found that females more often reported negative impacts than males in all domains—employment, family life, and mental and physical health. This disproportionate negative impact on females is consistent with prior public health emergencies [11], including the 2016 Zika and 2014 Ebola outbreaks [12]. Among US women, this has been described in several areas, including the health care workforce, reproductive health, drug development, gender-based violence, and mental health [13]. It is both notable and concerning that nearly 1123 (75%) of younger adults (aged 18-29 years) reported negative impacts on their mental health, which was higher than for any other group. The younger end of this range captures members of Generation Z, who are more likely to report poor mental health compared to prior generations [14,15]. However, they are also more likely to receive mental health therapy or treatment [14] and, therefore, may accept interventions to address the negative mental health consequences of the COVID-19 pandemic. Further, we found that negative impacts on employment were more commonly reported among Black participants. These findings highlight the breadth of negative impacts of this pandemic in our community and reveal the disproportionate impact experienced by certain subgroups that should be targeted in future intervention efforts.

Our study population had a much higher vaccination rate compared to Colorado overall and the general US population. Over 95% of our survey participants are fully vaccinated compared to 76% of adults throughout Colorado [16]. Vaccination directly reduces the likelihood of infection and severity of disease, but it also has an indirect effect on society via reduced viral transmission and herd immunity. Because of this impact on others, getting vaccinated is considered a prosocial behavior [17-19]. Being a participant in a biobank has also been positively associated with prosocial behavior, as the individuals who participate in biobanks tend to be motivated by furthering research for the greater good [20,21]. Since our study population only includes those who elected to be in the Biobank and additionally those who responded to the survey, these are likely individuals with high levels of prosocial behaviors, which likely explains the high vaccination rate.

COVID-19 has variable clinical presentations ranging from asymptomatic infections to severe symptoms that require hospitalization. We expected that COVID-19 patients identified in EHRs would be more likely to have severe COVID-19 and less likely to have asymptomatic infections than those captured by the survey [22,23]. However, we found that there was a slightly higher percentage of COVID-19 hospitalizations among survey cases compared to EHR cases. This unexpected result may be explained, in part, by the fact that individuals who were hospitalized with COVID-19 may be highly motivated to contribute to COVID-19 research by taking the survey. This likely includes individuals who went to non-UCHealth hospitals, which would not have been identified in EHRs. With respect to participant demographics, it is notable that a higher percentage of younger (18-29 years) and Hispanic/Latino COVID-19-positive cases were identified via EHRs versus the survey. This may, in part, be explained by lower survey response rates in these groups. Hispanic/Latino individuals may have been less likely to take the survey because of language barriers (the survey was only in English), limited internet access, or other structural barriers [24]. Lower participation among Hispanic individuals is consistent with observations in other outreach efforts [25] and is a limitation of the convenience survey design. Additionally, the Hispanic population in Colorado, as in many other states, had a higher incidence of COVID-19 infections, hospitalizations, and death [4,26-29], which may explain why they are more likely to be identified through EHRs.

### Conclusion

The combination of EHR and survey data provides a powerful opportunity to monitor and describe the ongoing effects of the COVID-19 pandemic in our communities. As the pandemic continues, there is a critical need for optimal COVID-19 case ascertainment in order to capture both mild and severe cases and monitor specific long-term outcomes, such as postacute sequelae of SARS-CoV-2 infection (PASC) or downstream breakthrough infections postvaccination. In an open health system, as is common in the United States, the development of a combined resource such as ours (with EHR and survey data) represents long-term potential for additional recruitment and follow-up as a critical complement to large-scale informatics-focused investigations, such as the National COVID Cohort Collaborative [30]. As the pandemic continues, we anticipate that resources such as the CCPM Biobank and other biobanks will continue to be a key resource for ongoing data collection relevant to population health monitoring during the era of COVID-19 and other emerging public health issues.

## Appendix

Multimedia Appendix 1 Characteristics of participants in the Biobank at the Colorado Center for Personalized Medicine Compared to the UC Health System.

Multimedia Appendix 2 COVID-19 survey instrument administered in 2020.

Multimedia Appendix 3 COVID-19 Survey Instrument Administered in 2021.

Multimedia Appendix 4 Data availability from the EHR and the survey.

Multimedia Appendix 5 COVID-19 specific encounter primary diagnoses used in addition to U07.1 to identify “EHR-confirmed case” from UC Health EHR data.

Multimedia Appendix 6 Specific encounter primary diagnoses used in conjunction with hospitalization, timing of hospitalization, and COVID-19 case definitions to identify “EHR-hospitalization” from UC Health EHR data.

Multimedia Appendix 7 Characteristics of Biobank participants by COVID-19 survey response.

## Abbreviations

CCPM: Colorado Center for Personalized Medicine
CDPHE: Colorado Department of Public Health and the Environment
EHR: electronic health record
ER: emergency room
GI: gastrointestinal
HDC: Health Data Compass
ICD-10: International Statistical Classification of Diseases, 10th revision
