# Supplementary material for: COVID-19 Surveillance in the Biobank at the Colorado Center for Personalized Medicine: Observational Study
Source: JMIR Public Health Surveill. 2022 Jun 13;8(6):e37327. doi: 10.2196/37327 (PMC9196874; doi:10.2196/37327)
Supplement: Multimedia Appendix 1 [file publichealth_v8i6e37327_app1.docx]

**Multimedia Appendix 1:** Characteristics of Participants in the Biobank at the Colorado Center for Personalized Medicine Compared to the UC Health System.

|  | **Biobank Participants*** | **UC Health**** |
| --- | --- | --- |
| **Characteristics** | **N=180,599** | **N=2,669,633** |
| **Sex** |  |  |
| Female | 60% | 54% |
| Male | 41% | 46% |
| Unknown | 0% | 0% |
| **Ethnicity** |  |  |
| Not Hispanic or Latino | 88% | 72% |
| Unknown | 3% | 15% |
| Hispanic or Latino | 9% | 13% |
| **Race** |  |  |
| White | 83% | 70% |
| Unknown | 10% | 22% |
| Black or African American | 4% | 6% |
| Asian | 2% | 2% |
| American Indian or Alaska Native | 0% | 0% |
| Native Hawaiian or Other Pacific Islander | 0% | 0% |
| **Age** |  |  |
| 18-29 | 13% | 19% |
| 30-39 | 22% | 19% |
| 40-49 | 17% | 16% |
| 50-59 | 16% | 15% |
| 60-69 | 17% | 15% |
| 70-79 | 12% | 10% |
| 80+ | 3% | 7% |
| Unknown | 0% | - |
| **Biobank at the CCPM enrollment as of 05/31/2021* | | |
| ***UC Health patient population (>= 18 years of age) accessed from TriNetX, Data updated 6/24/2021* | | |
