# Supplementary figures and images for: COVID-19 Surveillance in the Biobank at the Colorado Center for Personalized Medicine: Observational Study
Source: JMIR Public Health Surveill. 2022 Jun 13;8(6):e37327. doi: 10.2196/37327 (PMC9196874; doi:10.2196/37327)

**Multimedia Appendix 2:** COVID-19 Survey Instrument Administered in 2020**
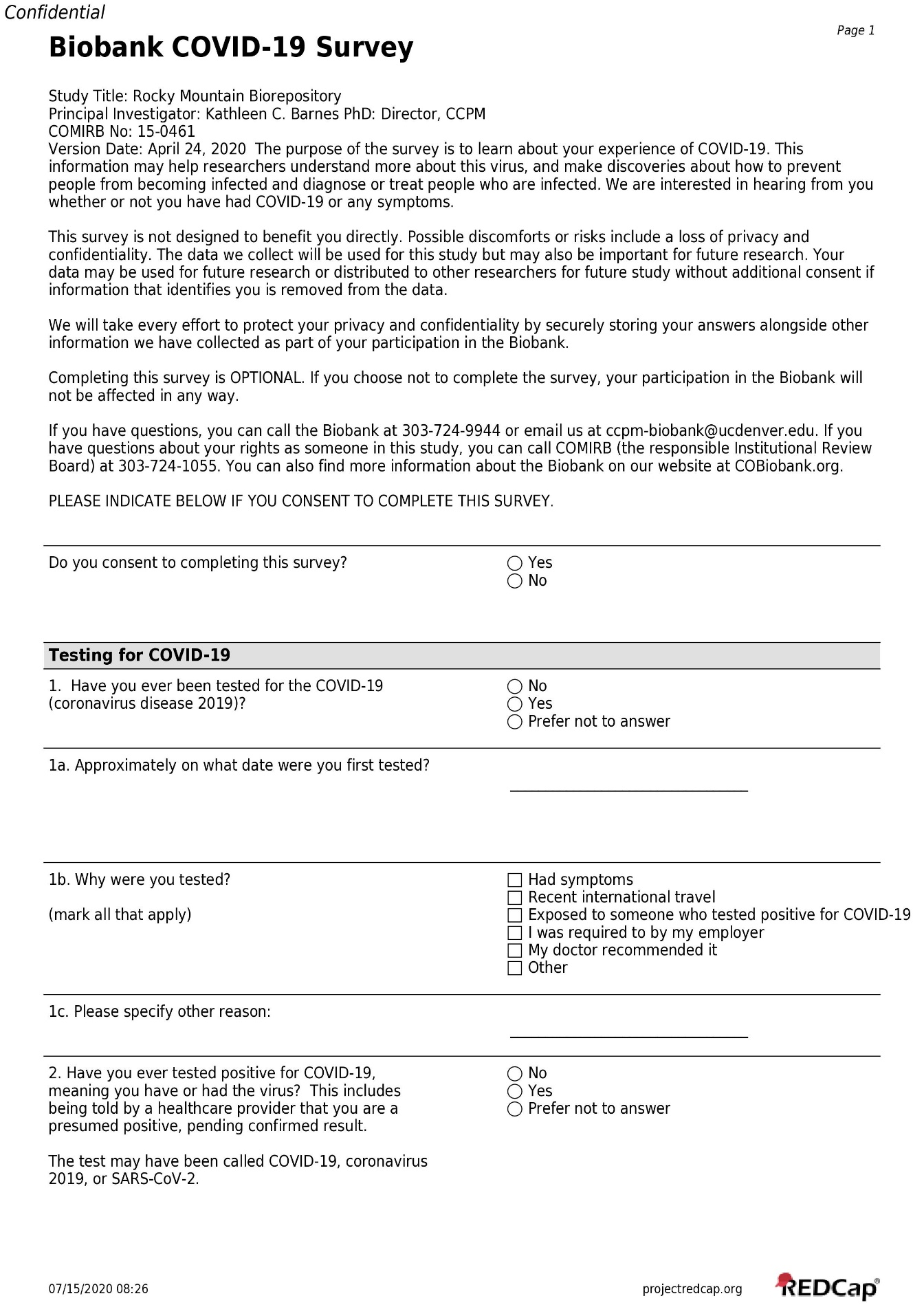
**

**
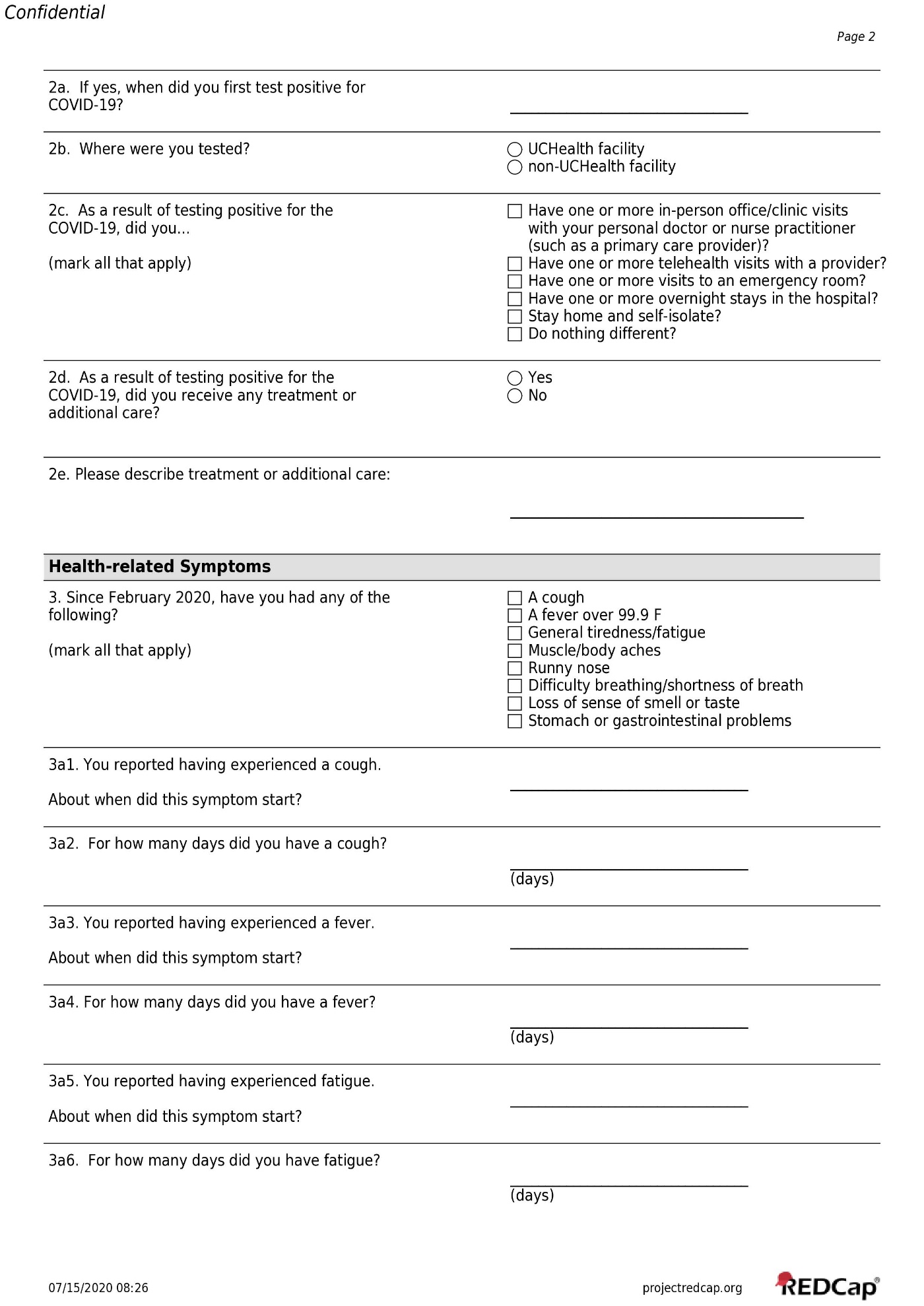

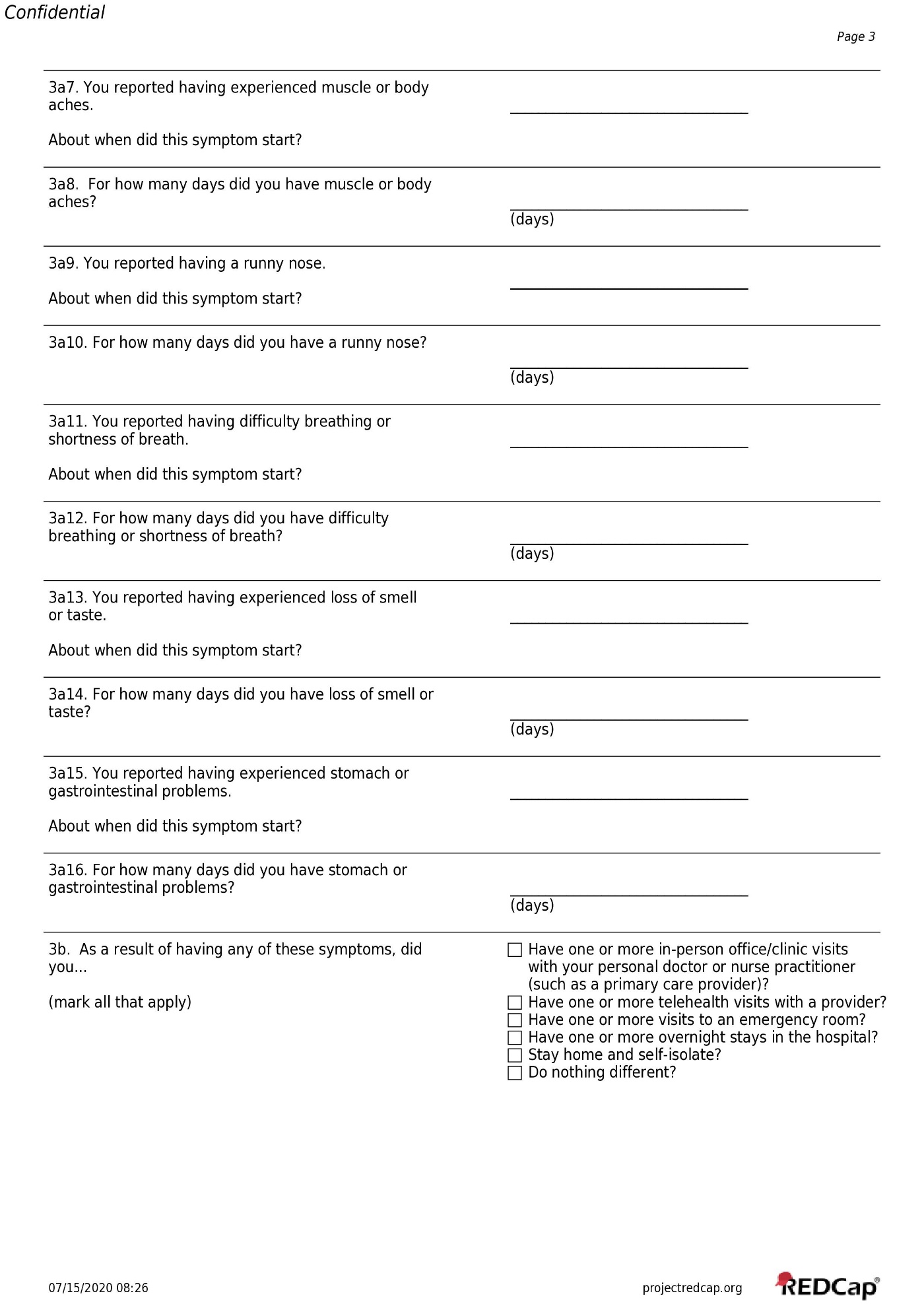

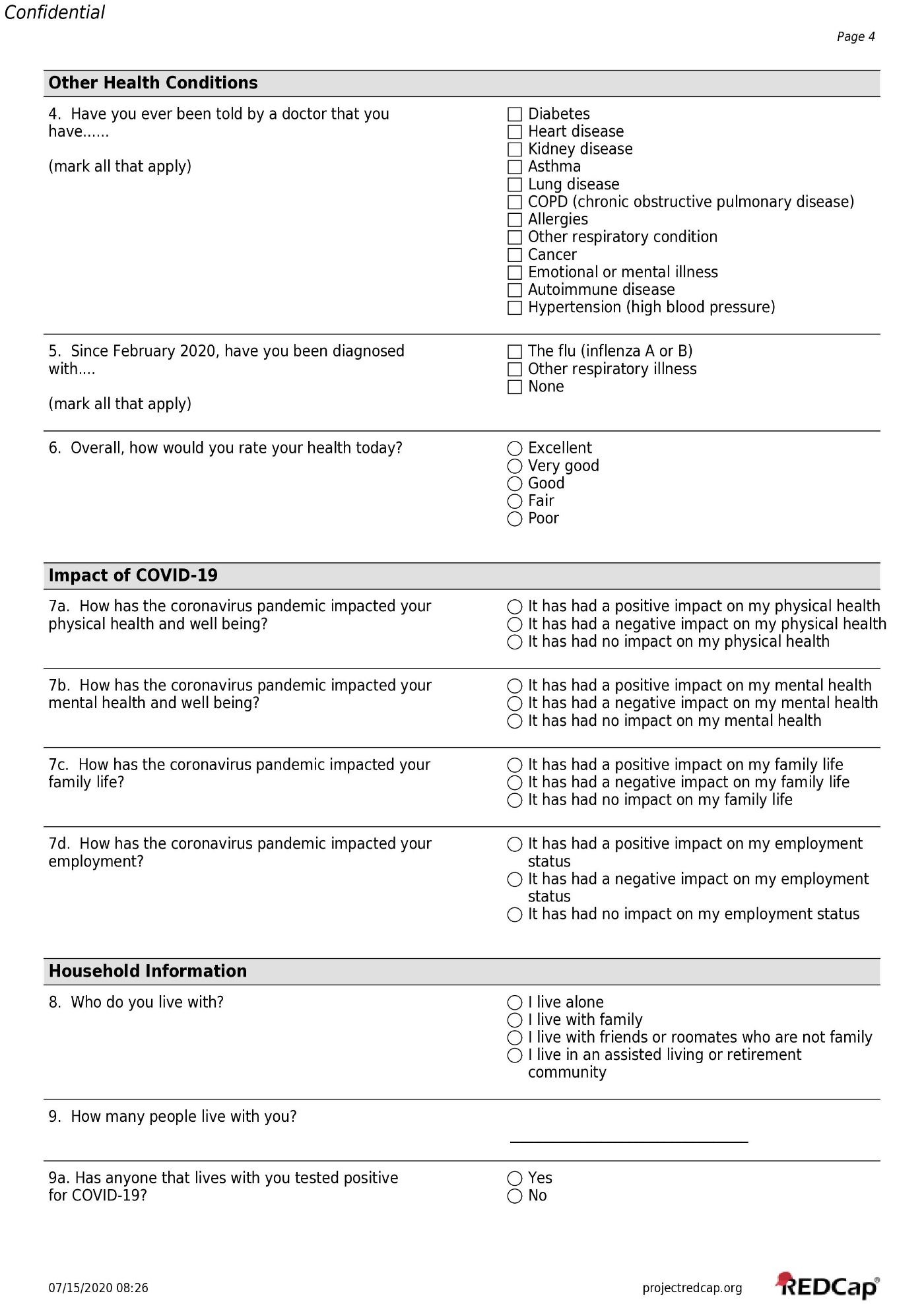

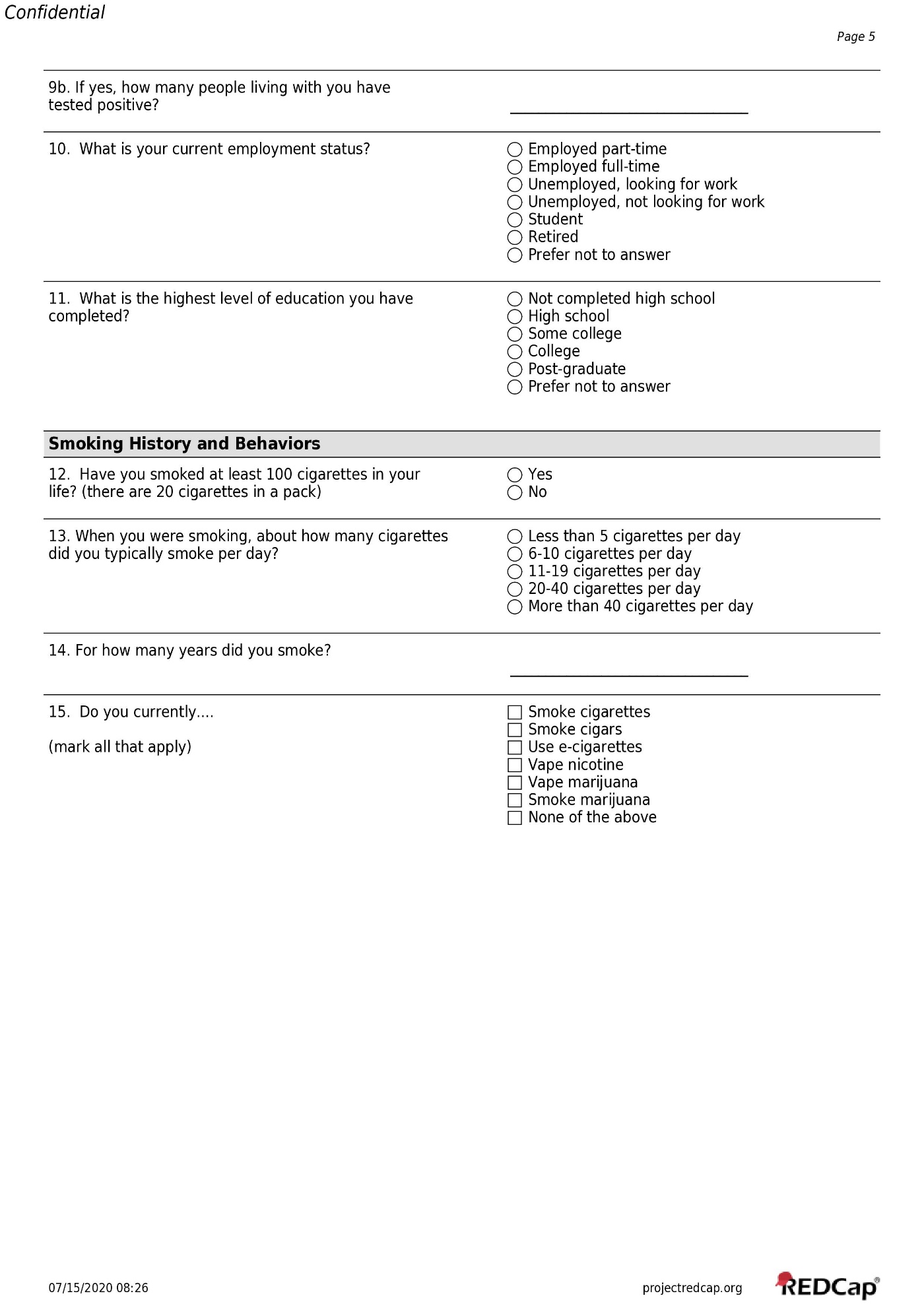
**

Supplement: Multimedia Appendix 2 [file publichealth_v8i6e37327_app2.docx]

**Multimedia Appendix 3:** COVID-19 Survey Instrument Administered in 2021**
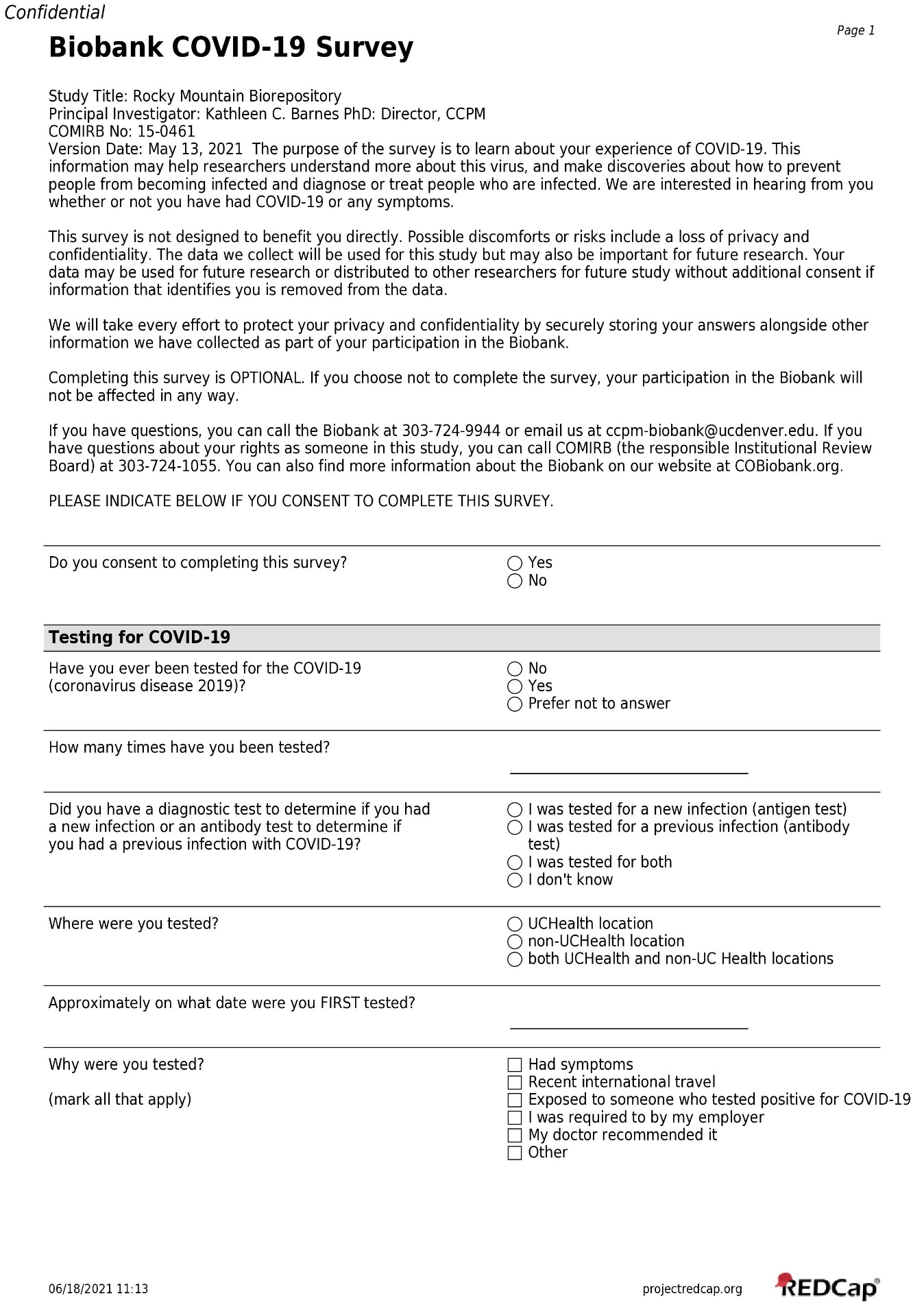
**

**
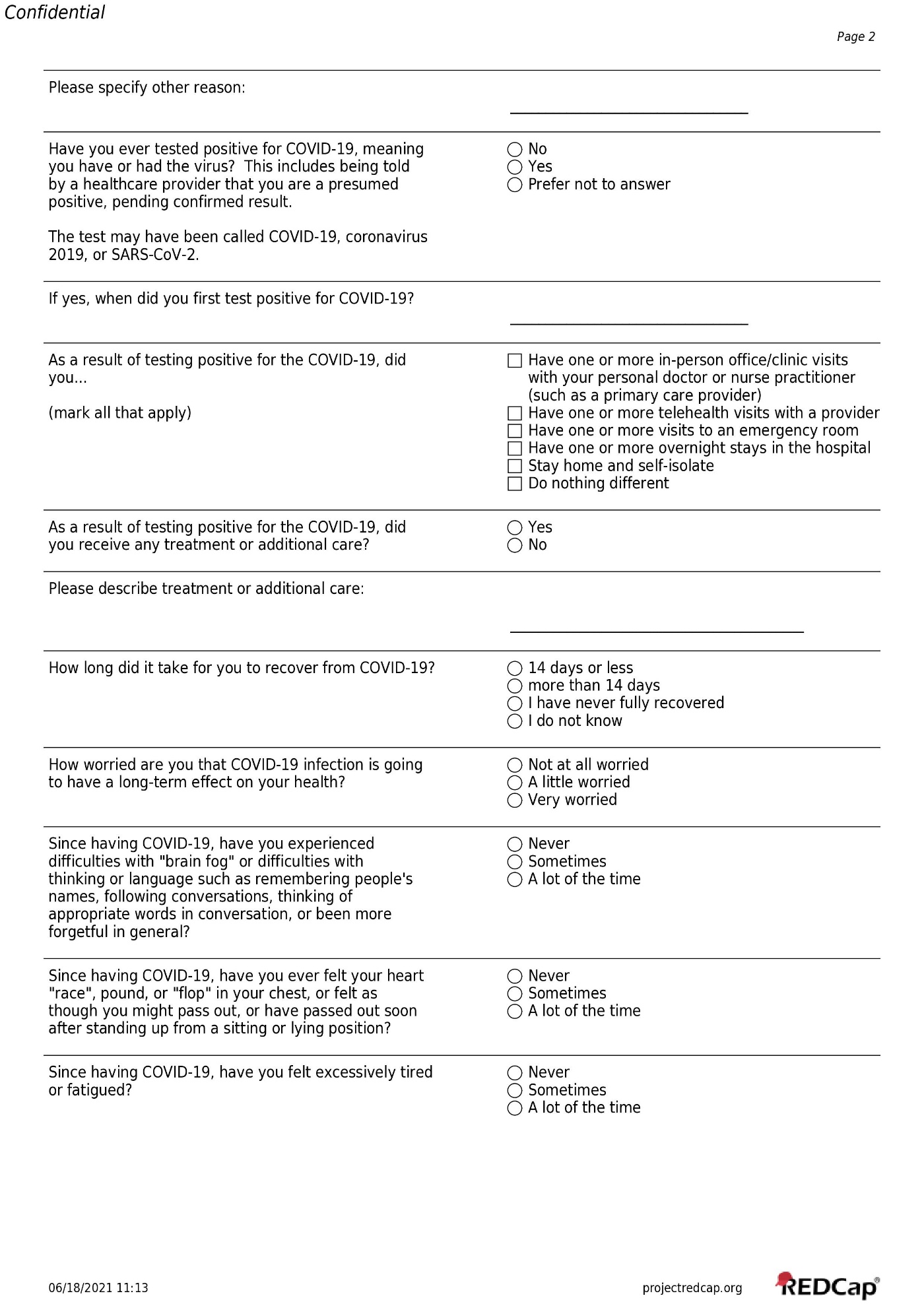

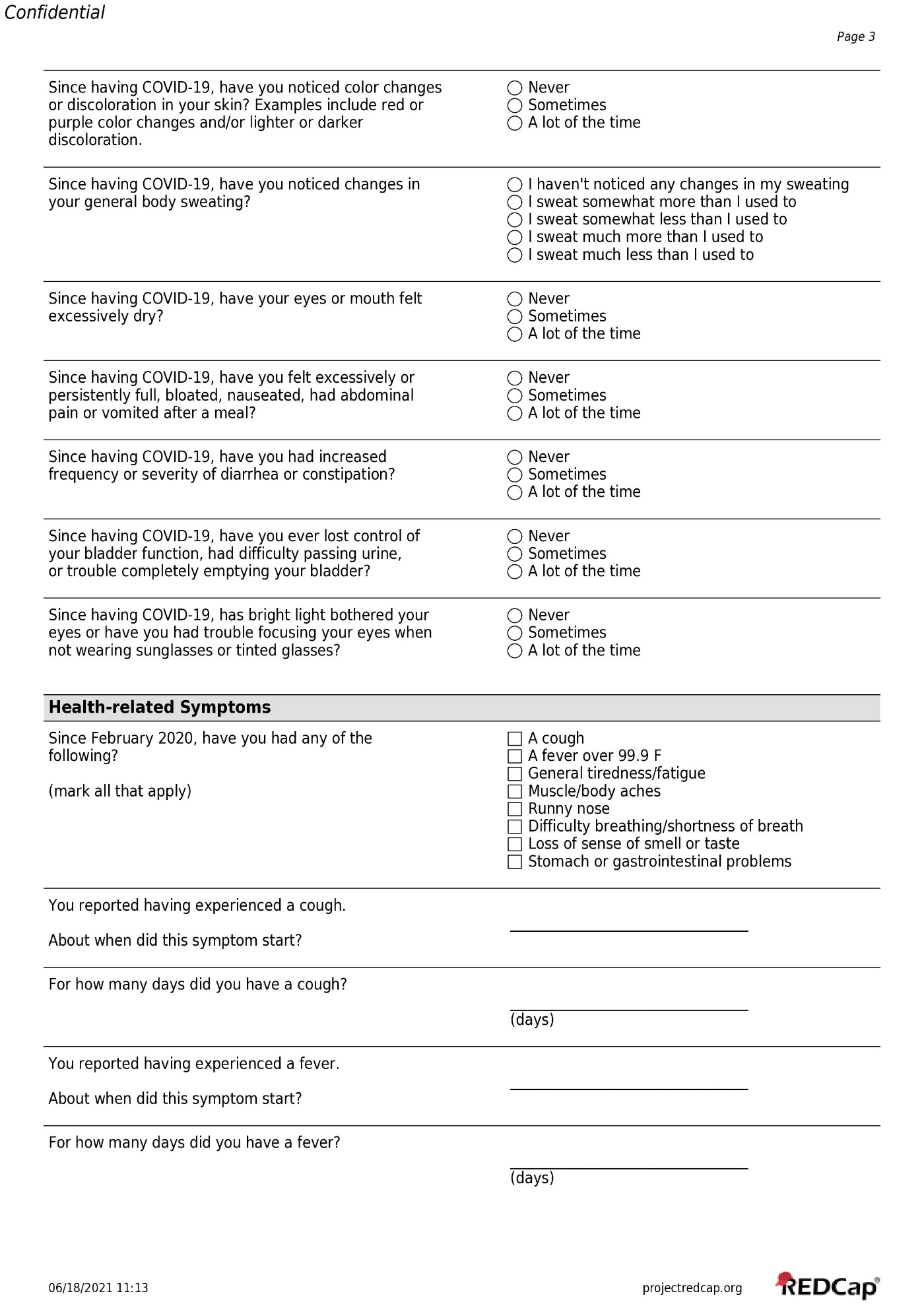

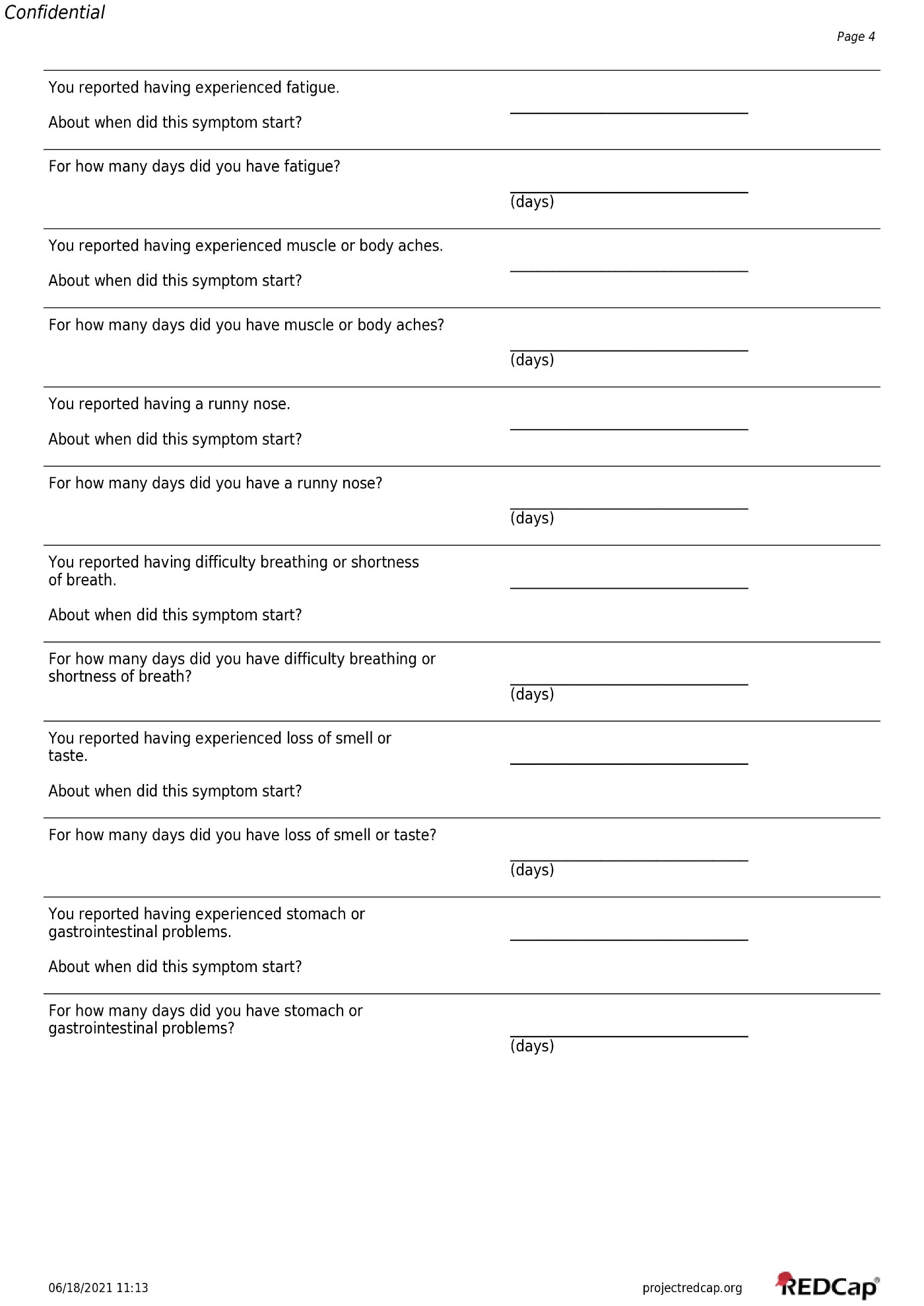

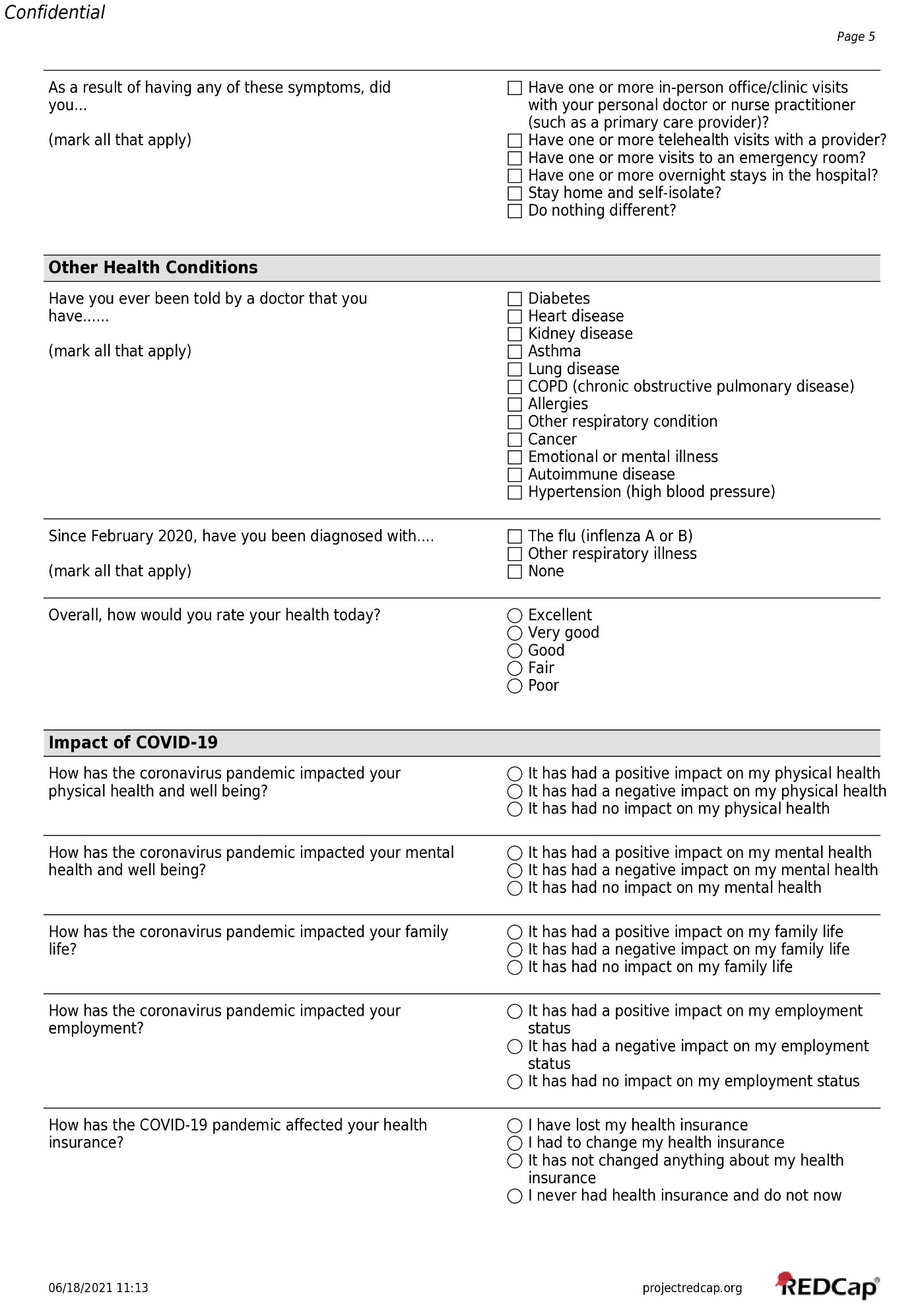

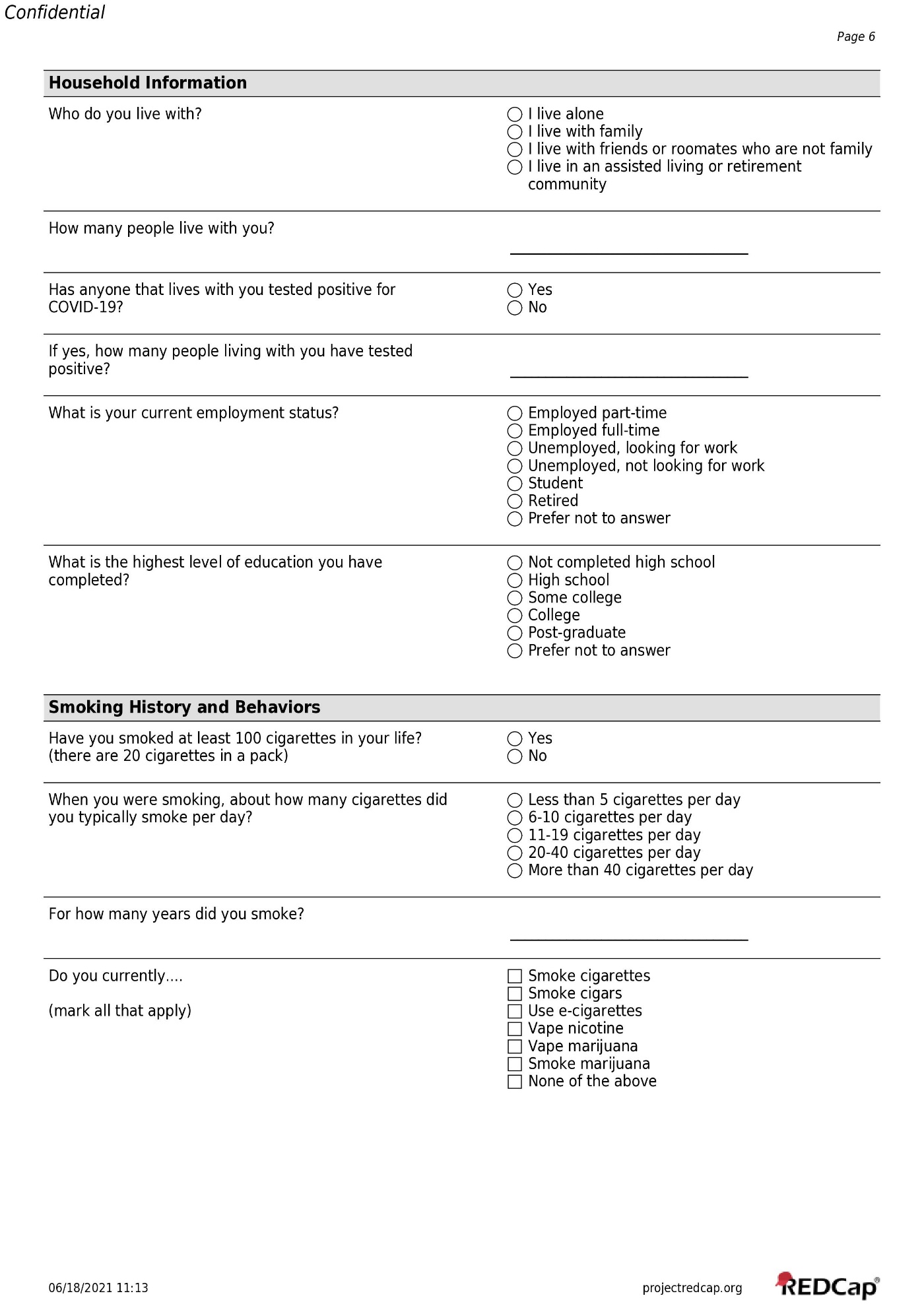

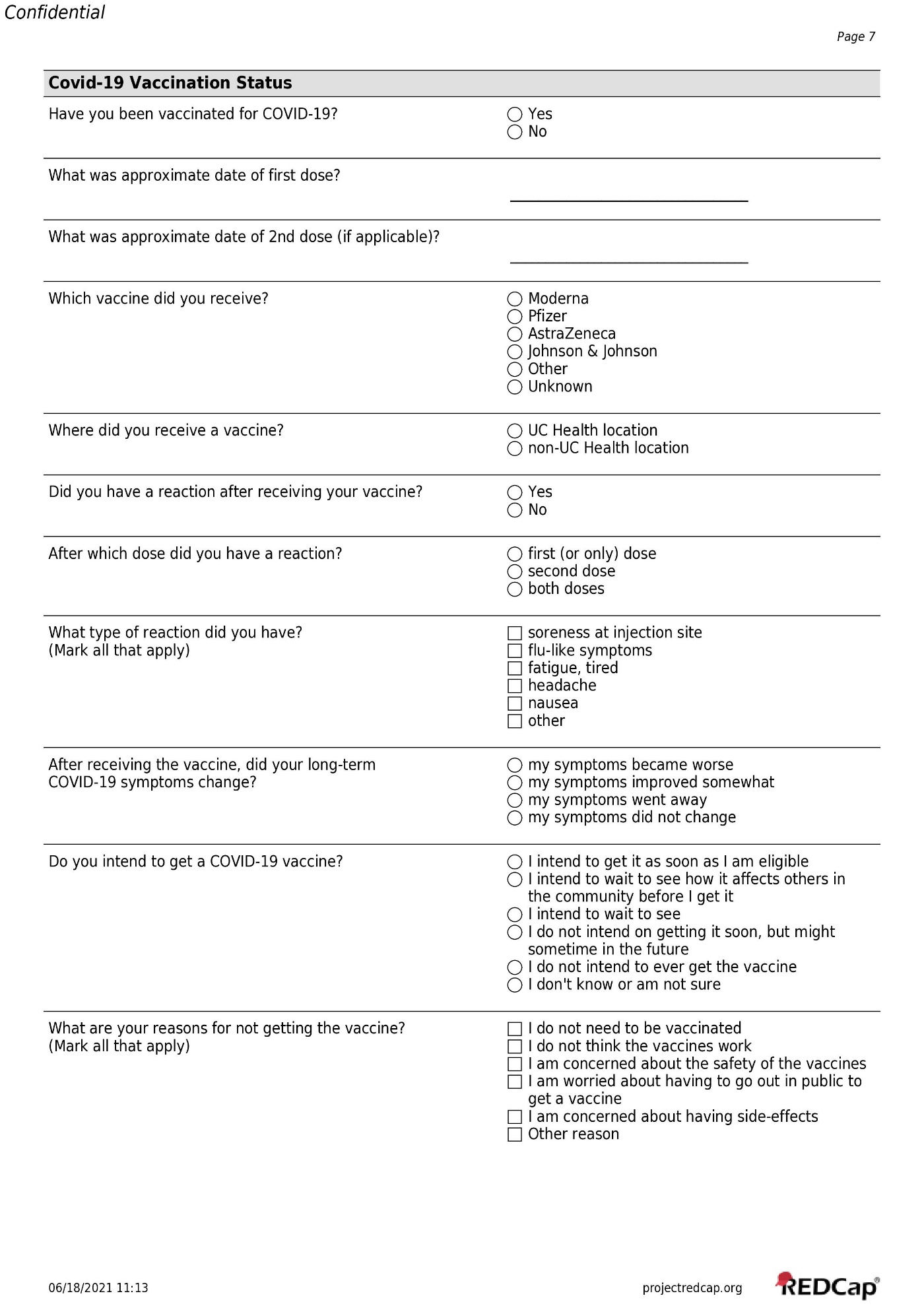

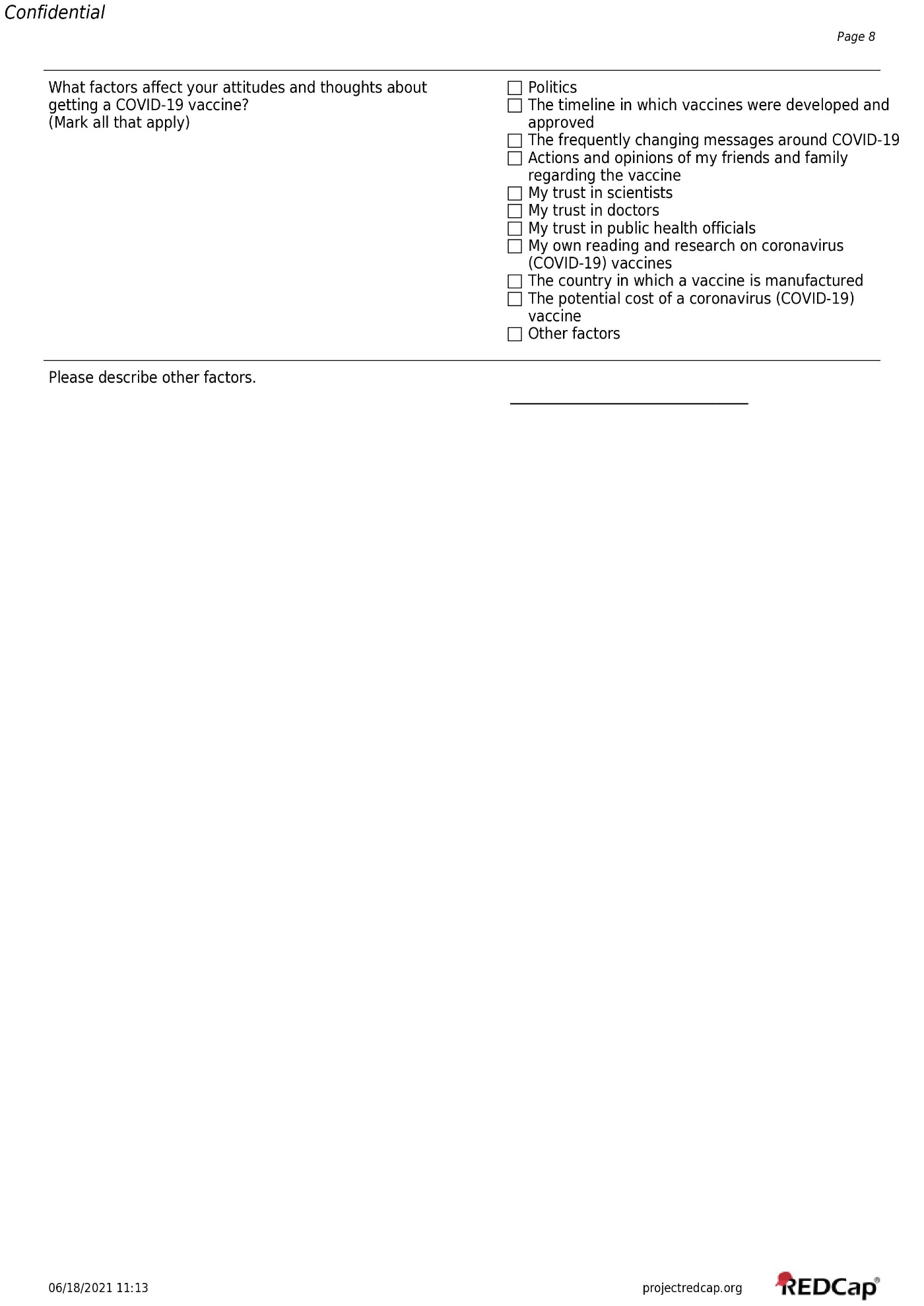
**

Supplement: Multimedia Appendix 3 [file publichealth_v8i6e37327_app3.docx]
