# Supplementary material for: COVID-19 Surveillance in the Biobank at the Colorado Center for Personalized Medicine: Observational Study
Source: JMIR Public Health Surveill. 2022 Jun 13;8(6):e37327. doi: 10.2196/37327 (PMC9196874; doi:10.2196/37327)
Supplement: Multimedia Appendix 4 [file publichealth_v8i6e37327_app4.docx]

**Multimedia Appendix 7:** Data availability from the EHR and the survey
