# Supplementary material for: COVID-19 Surveillance in the Biobank at the Colorado Center for Personalized Medicine: Observational Study
Source: JMIR Public Health Surveill. 2022 Jun 13;8(6):e37327. doi: 10.2196/37327 (PMC9196874; doi:10.2196/37327)
Supplement: Multimedia Appendix 5 [file publichealth_v8i6e37327_app5.docx]

**Multimedia Appendix 4:** COVID-19 specific encounter primary diagnoses used in addition to U07.1 to identify “EHR-confirmed case” from UC Health EHR data.

| "2019 novel coronavirus disease (COVID-19)", |
| --- |
| "Acute respiratory disease due to COVID-19 virus", |
| "Acute respiratory distress syndrome (ARDS) due to COVID-19 virus", |
| "COVID-19 virus detected", |
| "Encephalopathy due to COVID-19 virus", |
| "Gastroenteritis due to COVID-19 virus", |
| "Myocarditis due to COVID-19 virus", |
| "Otitis media due to COVID-19 virus", |
| "Pneumonia due to COVID-19 virus", |
| "Real time reverse transcriptase PCR positive for COVID-19 virus", |
| "Upper respiratory tract infection due to COVID-19 virus" |
