# Supplementary material for: COVID-19 Surveillance in the Biobank at the Colorado Center for Personalized Medicine: Observational Study
Source: JMIR Public Health Surveill. 2022 Jun 13;8(6):e37327. doi: 10.2196/37327 (PMC9196874; doi:10.2196/37327)
Supplement: Multimedia Appendix 6 [file publichealth_v8i6e37327_app6.docx]

**Multimedia Appendix 5:** Specific encounter primary diagnoses used in conjunction with hospitalization, timing of hospitalization, and COVID-19 case definitions to identify “EHR-hospitalization” from UC Health EHR data.

| "COVID-19", |
| --- |
| "Other viral diseases complicating childbirth", |
| "Viral infection, unspecified", |
| "Pneumonia due to other specified infectious organisms", |
| "Sepsis due to COVID-19 (HC code)", |
| "Other viral pneumonia", |
| "Nausea and vomiting", |
| "Cerebral infarction due to unspecified occlusion or stenosis of right posterior cerebral artery", |
| "Acute on chronic systolic (congestive) heart failure", |
| "Acute on chronic combined systolic (congestive) and diastolic (congestive) heart failure", |
| "Other transient cerebral ischemic attacks and related syndromes", |
| "Acute kidney failure with tubular necrosis", |
| "Acute on chronic respiratory failure, unspecified whether with hypoxia or hypercapnia (HC code)", |
| "Acute embolism and thrombosis of left femoral vein", |
| "AMS (altered mental status)", |
| "Other coronavirus as the cause of diseases classified elsewhere", |
| "Single subsegmental pulmonary embolism without acute cor pulmonale", |
| "Other specified sepsis", |
| "Other viral diseases complicating pregnancy, third trimester", |
| "Other pulmonary embolism without acute cor pulmonale", |
| "Acute respiratory failure with hypoxia", |
| "Other encephalopathy", |
| "Other diseases of pharynx", |
| "Heart failure, unspecified", |
| "Acute embolism and thrombosis of right iliac vein", |
| "Acute pulmonary embolism without acute cor pulmonale, unspecified pulmonary embolism type (HC code)", |
| "Saddle embolus of pulmonary artery with acute cor pulmonale", |
| "Contact with and (suspected) exposure to other viral communicable diseases", |
| "Acute combined systolic (congestive) and diastolic (congestive) heart failure", |
| "Hypoxemia", |
| "Acute on chronic diastolic (congestive) heart failure", |
| "Shortness of breath", |
| "Other pulmonary embolism with acute cor pulmonale", |
| "Pneumonia, unspecified organism", |
| "Suspected COVID-19 virus infection", |
| "Acute respiratory distress syndrome", |
| "Acute upper respiratory infection, unspecified", |
| "Sepsis (HC code)", |
| "ST elevation (STEMI) myocardial infarction involving left circumflex coronary artery", |
| "Non-ST elevation (NSTEMI) myocardial infarction", |
| "COVID-19 virus infection", |
| "Cerebral infarction due to embolism of other cerebral artery", |
| "Pneumonia due to COVID-19 virus", |
| "Severe sepsis with septic shock (CODE) (HC code)", |
| "Embolism and thrombosis of iliac artery", |
| "Acute and chronic respiratory failure with hypoxia", |
| "Hypoxia", |
| "Cerebral infarction due to unspecified occlusion or stenosis of left carotid arteries", |
| "ST elevation (STEMI) myocardial infarction involving other coronary artery of inferior wall", |
| "Acute respiratory failure with hypoxia (HC code)", |
| "Cerebral infarction due to embolism of right middle cerebral artery", |
| "ST elevation (STEMI) myocardial infarction involving other coronary artery of anterior wall", |
| "Other specified respiratory disorders", |
| "Other cerebral infarction", |
| "Unspecified acute lower respiratory infection", |
| "Other chest pain", |
| "Viral pneumonia, unspecified", |
| "Other viral infections of unspecified site", |
| "Sepsis, unspecified organism", |
| "Other viral diseases complicating pregnancy, first trimester", |
| "Multiple subsegmental pulmonary emboli without acute cor pulmonale", |
| "Cerebral infarction, unspecified", |
| "Transient cerebral ischemic attack, unspecified", |
| "Diseases of the respiratory system complicating childbirth", |
| "Cerebral infarction due to unspecified occlusion or stenosis of unspecified carotid artery" |
