# Supplementary material for: COVID-19 Surveillance in the Biobank at the Colorado Center for Personalized Medicine: Observational Study
Source: JMIR Public Health Surveill. 2022 Jun 13;8(6):e37327. doi: 10.2196/37327 (PMC9196874; doi:10.2196/37327)
Supplement: Multimedia Appendix 7 [file publichealth_v8i6e37327_app7.docx]

**Multimedia Appendix 6:** Characteristics of Biobank participants by COVID-19 survey response.

|  | **Total Biobank*** | **Response** | **Non-Response** | ***P-value*** |
| --- | --- | --- | --- | --- |
| **Characteristics** | **N=180,599** | **N=25,075** | **N=155,524** |  |
| **Age, mean (SD)** | 49.5 (17.0) | 55.0 (15.8) | 48.6 (17.0) | <.001 |
| **Age, n (%)** |  |  |  | <.001 |
| 18-29 | 23790 (13.2%) | 1504 (6.0%) | 22286 (14.3%) |  |
| 30-39 | 38825 (21.5%) | 3893 (15.5%) | 34932 (22.5%) |  |
| 40-49 | 30872 (17.1%) | 3824 (15.3%) | 27048 (17.4%) |  |
| 50-59 | 29248 (16.2%) | 4472 (17.8%) | 24776 (15.9%) |  |
| 60-69 | 30722 (17.0%) | 6180 (24.6%) | 24542 (15.8%) |  |
| 70-79 | 21025 (11.6%) | 4357 (17.4%) | 16668 (10.7%) |  |
| 80+ | 5991 (3.3%) | 836 (3.3%) | 5155 (3.3%) |  |
| Missing | 126 (0.1%) | 9 (0.0%) | 117 (0.1%) |  |
| **Sex, n (%)** |  |  |  | <.001 |
| Female | 107402 (59.5%) | 15695 (62.6%) | 91707 (59.0%) |  |
| Male | 73061 (40.5%) | 9368 (37.4%) | 63693 (41.0%) |  |
| Unknown | 136 (0.1%) | 12 (0.0%) | 124 (0.1%) |  |
| **Race-Ethnicity, n (%)** |  |  |  | <.001 |
| Non-Hispanic White | 141765 (78.5%) | 21917 (87.4%) | 119848 (77.1%) |  |
| Non-Hispanic Black | 6816 (3.8%) | 308 (1.2%) | 6508 (4.2%) |  |
| Hispanic | 16283 (9.0%) | 1272 (5.1%) | 15011 (9.7%) |  |
| Asian | 3628 (2.0%) | 329 (1.3%) | 3299 (2.1%) |  |
| American Indian and Alaska Native | 513 (0.3%) | 47.0 (0.2%) | 466 (0.3%) |  |
| Native Hawaiian and Other Pacific Islander | 288 (0.2%) | 15 (0.1%) | 273 (0.2%) |  |
| Non-Hispanic Other | 11180 (6.2%) | 1178 (4.7%) | 10002 (6.4%) |  |
| Unknown | 126 (0.1%) | 9 (0.0%) | 117 (0.1%) |  |
| **Genetic Data** |  |  |  | <.001 |
| N | 147647 (81.8%) | 20194 (80.5%) | 127453 (82.0%) |  |
| Y | 32952 (18.2%) | 4881 (19.5%) | 28071 (18.0%) |  |
| **Medican Income**, mean (SD)** | 75000 (24000) | 76700 (23800) | 74700 (24000) | <.001 |
| **Percent Above Bachelors Education**, mean (SD)** | 43.7 (17.4) | 46.6 (16.6) | 43.3 (17.5) | <.001 |
| **Biobank participants as of May 31, 2021, Responses to survey received by 10/31/2021* | | | | |
| ***Based on 3-digit zip code of residence* | | | | |
